# Knowledge About Individuals’ Interracial Friendships Is Systematically Associated With Mental Representations of Race, Traits, and Group Solidarity

**DOI:** 10.1177/01461672211024118

**Published:** 2021-06-19

**Authors:** Jonas R. Kunst, Ivuoma N. Onyeador, John F. Dovidio

**Affiliations:** 1 University of Oslo, Norway; 2 Yale University, New Haven, CT, USA; 3 Northwestern University, Evanston, IL, USA

**Keywords:** extended contact, interracial contact, mental representation, racial perception, social categorization

## Abstract

Individuals with other-race friends are perceived to identify less strongly with their racial in-group than are individuals with same-race friends. Using the reverse-correlation technique, we show that this effect goes beyond perceptions of social identification, influencing how people are mentally represented. In four studies with Black and White American participants, we demonstrate a “racial assimilation effect”: Participants, independent of their own race, represented both Black and White targets with other-race friends as phenotypically more similar to the respective racial out-group. Representations of targets with racial out-group friends were subsequently rated as more likely to engage in social action supportive of the racial out-group. Out-group targets with other-race friends were represented more favorably than out-group targets with mostly same-race friends. White participants had particularly negative representations of in-group members with mostly Black friends. The present research suggests that individuals’ social networks influence how their race and associated traits are mentally represented.

Intergroup biases are shaped by the nature of social exchanges between groups as well as by the characteristics of members of different groups. Socially, cross-group friendships, both personally experienced (Davies et al., 2011) and exhibited by other in-group members (Wright et al., 1997), predict favorable intergroup attitudes. In terms of characteristics, facial appearance plays a critical role (Zebrowitz et al., 2013). People whose facial features are more prototypical of their group are characterized in ways more consistent with stereotypes of their group and are treated in ways more reflective of social biases toward the group (Maddox, 2004; Maddox & Perry, 2017). For instance, White Americans more strongly stereotype and negatively evaluate and treat Black Americans who have more phenotypically prototypical (“Afrocentric”) facial features (Blair et al., 2004; Livingston & Brewer, 2002). Although there are robust separate literatures on the effects of intergroup friendships and facial characteristics on intergroup relations, in this research, we identify a unique link that bridges these largely separate lines of work. Specifically, we test how beliefs about others’ intergroup friendships relate to intergroup responses by affecting the way people mentally represent members of in-groups and out-groups. We considered this issue in the context of race relations in the United States and from the perspectives of both White and Black Americans.

Understanding how mental representations of members of different groups systematically vary as a function of knowledge about their social relationships has important theoretical and practical implications. Theoretically, while information about others’ friendship networks has been demonstrated to influence impressions of members of other groups (e.g., perceptions of their group identification) in systematic ways (Cooley et al., 2018; Johnson & Ashburn-Nardo, 2014), we studied a mechanism—mental representations of others—that has, to the best of our knowledge, not been considered in this context but can have powerful effects on social perceptions. Mental images of members of different social groups play a major role in reactions to others, shaping responses at the earliest stages of impression formation (Todorov et al., 2015) and automatically activating stereotypes, attitudes, and feelings (Blair et al., 2004). Perceived prototypicality of facial appearance can independently influence responses through a category-based route by facilitating social categorization of an individual as a member of a different racial group (Maddox, 2004), which elicits a range of group-related impressions and expectations. However, as revealed more than 70 years ago in classic “new look” research (e.g., Bruner & Goodman, 1947), people’s perceptions are shaped by a range of influences, such as personal motives and values. Beliefs about others’ friendship networks may represent one such factor. Practically, despite the fact that many societies are rapidly becoming more racially diverse, close relations between members of different racial groups are quite rare (Cox & Jones, 2016). Because having friends mostly from one’s own racial group is the social default, providing information that others have diverse friendship networks can provide an implementable avenue for affecting intergroup relations.

Two of the four studies we conducted used the reverse-correlation procedure (Dotsch & Todorov, 2012), which is a data-driven method to tap people’s mental representations of social categories. This approach illuminates biases toward categories such as gender or race (see Brinkman et al., 2017 for a review). In terms of racial biases, research has shown that “criminals,” “welfare recipients,” and “terrorists” often are represented as more phenotypically similar to minority- than majority-group members (Brown-Iannuzzi et al., 2016; Dotsch et al., 2011; Kunst, Myhren, & Onyeador, 2018). However, the reverse-correlation technique is also sensitive to differences within members of both minority and majority groups (e.g., subtypes; Hinzman & Maddox, 2017). For instance, Hinzman and Maddox (2017) showed that Black targets with occupations and roles such as “athlete” or “rapper” were mentally represented as more phenotypically prototypical, whereas the exact opposite was true for White targets.

In our reverse-correlation task, over a series of trials, participants selected from two faces the one that they believed had mostly own-race friends, an equal number of own- and other-race friends, or mostly other-race friends to generate a composite face that best visually represented that characteristic. The third study aimed to demonstrate that the effects on perceived phenotypicality were not limited to skin tone but also reflected in changes in physiognomy. The fourth study involved representative samples of Black and White participants who judged the appearance and traits of the target individuals represented by these images.

Mental representations of individuals are not static but “dynamically and probabilistically reconstructed” (Freeman & Ambady, 2011, p. 23). Both higher level cognitive states (e.g., knowledge about an individual’s social environment) and lower level cues (e.g., skin tone and physiognomy) can influence the way an individual is mentally represented. Group prototypes embody the attributes that define the group and make it distinctive from other groups (also see Hogg et al., 2017; Turner et al., 1987). Thus, to the extent that Black and White targets have mainly in-group friends—the social default in the United States—they will likely be seen as highly representative of their group and will be mentally represented as highly phenotypically prototypical. Specifically, in all studies, we predicted that Black targets would be mentally represented and perceived as having a more Afrocentric (and less Eurocentric) appearance when they are believed to have primarily Black friends compared with either an equal number of Black and White friends or mostly White friends. Similarly, we anticipated that White targets who primarily have White friends would be mentally represented as more Eurocentric (and less Afrocentric) in appearance compared with White targets with a substantial number of Black friends. Given that race-related mental representations often activate stereotypic expectations (Blair et al., 2002; Maddox, 2004; Todorov et al., 2015), we predicted that perceptions of the target’s support for actions viewed as primarily benefiting Whites or Blacks would show a pattern similar to that predicted for racial phenotypicality. In addition, based on the responses of independent samples of participants, we examined how the mental representations of individuals, which were created based on information about target individuals’ friendship networks, differ on basic dimensions of person perception, including warmth, trustworthiness, threat, competence, and social class.

We hypothesized that, because social networks convey important information about a target’s representativeness of others’ group, information about the friendships of out-group and in-group members can systematically affect how these individuals are mentally represented—as prototypical in terms of physical appearance and in terms of impressions of them. We expected that in-group members with mostly out-group friends would be mentally represented in generally negative ways. Members of high-power groups, such as Whites in the present research, may show negativity toward such individuals because they may be perceived as disloyal defectors who ultimately weaken the cohesion and power of the in-group (Sidanius & Pratto, 1999; also see Kunst et al., 2019). In-group members having mostly out-group friends may be also judged negatively by members of low-power groups because these members may be perceived to be less identified with their in-group, thereby weakening it (Johnson & Ashburn-Nardo, 2014; also see Kaiser & Pratt-Hyatt, 2009). Conversely, we expected that racial out-group members with mostly same-race friends would be mentally represented as strongly prototypical of their group in terms of facial skin tone and physiognomy. Also, social categorization processes influence the ways people represent and perceive the affective expressions of others (Kawakami et al., 2017). Thus, we also anticipated that out-group targets would be mentally represented with negative facial expressions. We expected this to be particularly likely to occur when the out-group member has mostly friends within their group, which signals exclusionary intergroup orientations (Shelton & Richeson, 2005).

To test our predictions, we conducted two studies to create composite facial images using the reverse-correlation technique (Dotsch & Todorov, 2012). In two subsequent studies, we obtained evaluations of these images by independent raters. Black and White American participants were asked across multiple trials to select from pairs of Black (Study 1) or White (Study 2) faces the individual they believed to have mostly Black friends, mostly White friends, or an equal number of both. Importantly, we imposed random noise on each pair of images, making them look slightly different from each other (see Figure 1). Based on participants’ average responses to 300 trials, classification images were computed representing approximations of the average mental representation that participants had within each condition. Next, to statistically test our hypotheses, we extracted and compared the skin tone intensities of the classification images that participants had generated of the Black and White targets in the different friendship conditions of the first two studies (see Krosch & Amodio, 2014). We then tested whether differences in phenotypicality ratings would not only be observed in terms of skin tone but also in terms of physiognomy (Study 3). Finally, the classification images were rated on various dimensions by an independent, nationally representative sample of Black and White Americans who were unaware of how these images were produced and our hypotheses (Study 4). All analyses were conducted in R 3.4.3. The *p* values are Holm-corrected for multiple comparisons. Given that the use of aggregate images in reverse-correlation research may inflate Type 1 error rates (Cone et al., 2021), our set of studies included ratings of individual and aggregate images.

**Figure 1.**
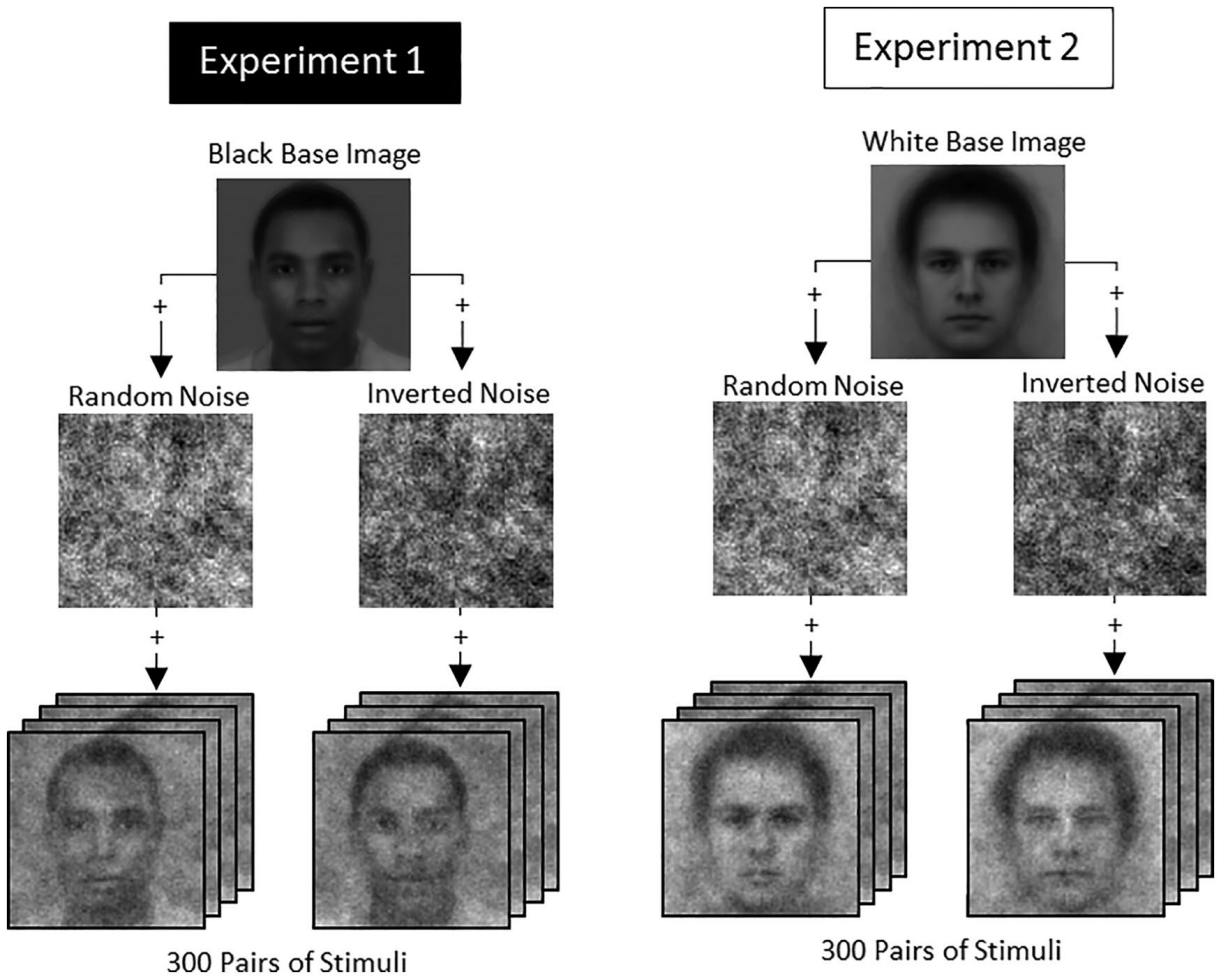
Reverse-correlation stimuli creation in Studies 1 and 2.

## Study 1: Skin Tone Test With Black Targets

The aim of Study 1 was to investigate whether mental representations of a Black individual’s race change as a function of the racial composition of his friendship circle. Our prediction was that, given the segregated nature of friendship networks representing the social default in the United States, Black targets believed to have primarily Black friends would be mentally represented as having a darker skin tone than Black targets believed to have mostly White or an equal number of White and Black friends.

### Method

#### Participants

This research was approved by the institutional review board of the primary institution of the first author. In each study, participants were prescreened and, thus, not aware of being recruited based on their race. The data, code, and materials needed to conduct the analyses can be found at https://osf.io/ta7cv/. In line with previous reverse-correlation research where 20 to 30 participants per experimental cell typically were sufficient to observe large effects (e.g., Dotsch & Todorov, 2012), we recruited 91 Black Americans (*M_age_ =* 36.27, *SD_age_ =* 10.93; women = 51.6%) and 90 White Americans (*M_age_ =* 39.09, *SD_age_ =* 12.00; women = 52.2%) via Amazon Mechanical Turk, who received US$3 for participation.

#### Procedure

We randomly assigned participants to one of the three conditions. In each condition, the participants completed a horizontal two-image, forced-choice reverse-correlation task (Dotsch & Todorov, 2012). Specifically, participants were told that they would see a series of image pairs of Black American individuals and were—dependent on condition—asked to select the individual whom they believed has (a) “mostly African American friends,” (b) “an equal number of White American and African American friends,” or (c) “mostly White American friends.” Participants completed 300 such trials presented in randomized order. Stimuli were created using the *rcicr* package (Dotsch, 2016) and the default noise pattern parameters. Random noise patterns were superimposed on a base image (averaged Black American male faces from the Chicago Face Database; Ma et al., 2015) to create the stimuli (see Figure 1).

In this study (and in Study 2), our dependent measure of facial appearance was skin tone because it is a distinguishing prototypical feature of Black and White racial groups in the United States (Maddox & Perry, 2017) and can be objectively scored using pixel intensity analysis.

#### Pixel intensity analysis

We extracted the mean pixel intensities from the classification images generated by each participant, which were masked such that areas that did not show the skin of the target (i.e., hair and regions outside the face) were excluded from the analysis. This left an area of interest of 108,141 pixels. These scores were standardized to facilitate interpretation. Higher scores represented a lighter skin tone.

### Results

**Figure 2.**
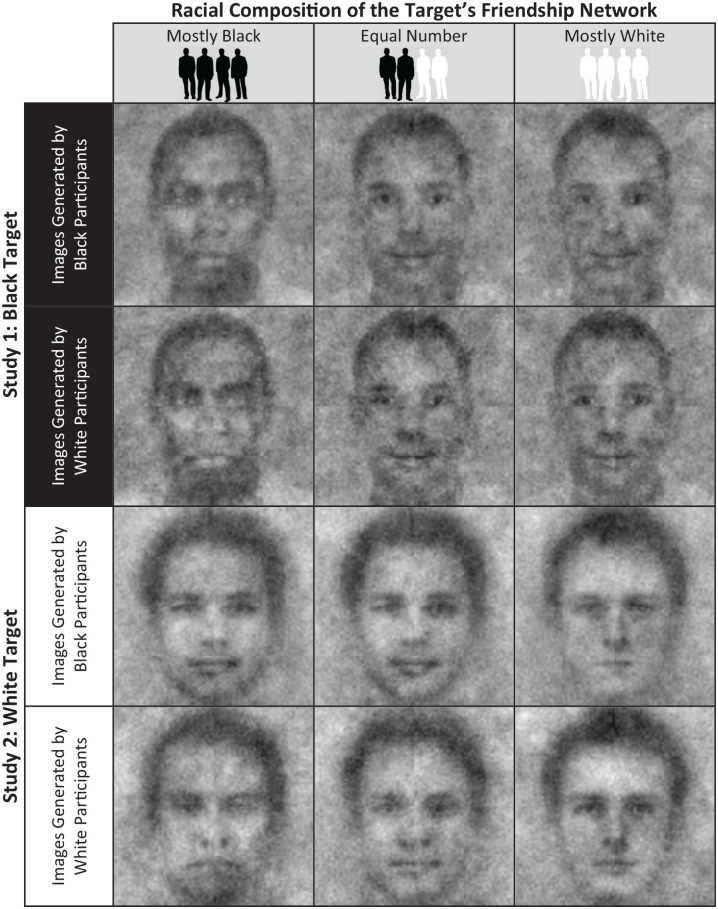
Black and White participants’ mental representations of Black and White targets as a function of the target’s friendship networks in Studies 1 and 2.

**Figure 3.**
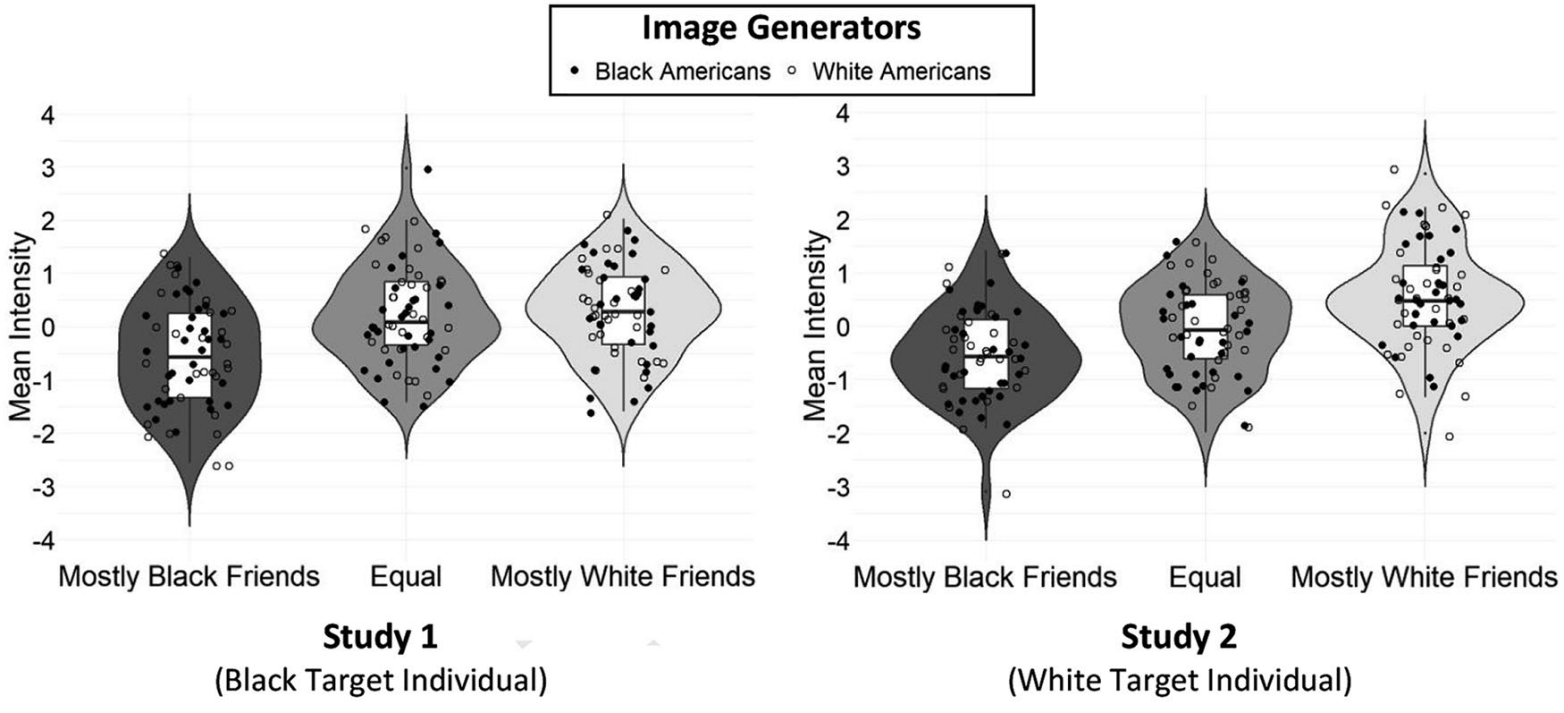
Pixel analyses are presented for Studies 1 and 2. *Note.* Higher values indicate lighter pixels in the classification images. Estimates are *z*-scored.

The first study generated classification images of Black targets whose friendship networks differed in terms of their racial composition (see upper half of Figure 2). A 3 (friendship condition) × 2 (reverse-correlation image generator race) ANOVA was conducted with the pixel intensities of the individual classification images that participants had generated of the Black targets as the dependent measure. As predicted and as displayed in Figure 3, the pixel intensities of the images varied as a function of the friendship condition to which participants responded, *F*(2, 175) = 5.34, *p =* .006, $\eta_{p}^{2}$ = .14. Relative to the Black target with mostly Black friends, *M = −*.53, *SE* = .12, the images of Black targets with partly (i.e., an equal number), *M =* .23, *SE* = .12, and mostly White friends, *M =* .29, *SE* = .12, were significantly lighter (see Table 1). Images of the latter two targets did not differ significantly. Neither the main effect of the race of participants who generated the images, *F*(1, 175) = .26, *p =* .614, $\eta_{p}^{2}$ < .01, nor its interaction with the friendship manipulation, *F*(2, 175)= .57, *p =* .569, $\eta_{p}^{2}$ = .01, were significant.

**Table 1.** Contrast Between the Different Conditions Across Studies.

Study / variable	Mostly own-race vs. equal number of own- and other-race				Mostly own-race vs. mostly other-race				Equal number of own- and other- race vs. mostly other race			
	*t/z* ^a^	*df*	*p*	*d/d_rm_* ^b^	*t/z* ^a^	*df*	*p*	*d/d_rm_* ^b^	*t/z* ^a^	*df*	*p*	*d/d_rm_* ^b^
Studies 1 and 2: Pixel intensities												
Black target	−4.49	175	<.001	0.68	−4.80	175	<.001	0.73	0.33	175	.734	0.05
White target	−3.60	177	<.001	0.54	−6.48	177	<.001	0.97	−2.89	177	.004	0.43
Study 3: Physiognomy ratings^c^												
Black target												
Afrocentrism	5.79	NA	<.001	0.60	6.12	NA	<.001	0.70	1.02	NA	.309	0.10
Eurocentrism	−5.94	NA	<.001	0.58	−6.94	NA	<.001	0.75	−1.82	NA	.068	0.16
White target												
Afrocentrism	3.81	NA	<.001	0.36	3.70	NA	<.001	0.43	0.66	NA	.512	0.07
Eurocentrism	−1.60	NA	.135	0.14	−2.93	NA	.010	0.33	−1.83	NA	.135	0.18
Study 4												
Black target												
Afrocentrism ratings	15.60	211	<.001	1.94	14.68	208	<.001	1.84	−1.05	369	.293	0.10
Black social action	4.03	192	<.001	0.55	4.05	223	<.001	0.49	−0.50	351	.619	0.05
Eurocentrism ratings	−13.68	264	<.001	1.51	−13.08	231	<.001	1.54	−0.36	333	.719	0.04
White social action	−10.21	298	<.001	1.06	−9.75	299	<.001	1.02	0.51	367	.610	0.05
White target												
Afrocentrism ratings	−9.32	211	<.001	1.17	−12.96	209	<.001	1.63	−4.74	369	<.001	0.46
Black social action	−8.26	192	<.001	1.09	−8.14	223	<.001	0.98	1.21	351	.226	0.12
Eurocentrism ratings	14.51	263	<.001	1.60	19.53	231	<.001	2.31	7.08	334	<.001	0.71
White social action	9.76	299	<.001	1.02	15.27	300	<.001	1.59	5.95	367	<.001	0.57

*Note*. NA = Not applicable.

a *Z*-test is presented instead of *t*-test when degrees of freedom exceeded 4,000.

b For repeated measures designs, *d_rm_* is reported.

c Controlling for pixel intensities from Studies 1 and 2.

### Discussion

Supportive of our hypothesis, participants mentally represented Black targets having primarily Black friends with a darker skin tone than Black targets having mostly or partly White friends. Having obtained initial support for our hypothesis, in the next study, we tested whether a corresponding pattern of results would be observed with White targets.

## Study 2: Skin Tone Test With White Targets

We aimed to replicate the results from the first study, this time with a White target. Mirroring Study 1, our prediction was that the White target believed to have mainly White friends would be mentally represented as having a lighter skin tone than the White targets believed to have mostly Black friends or an equal number of Black and White friends.

### Method

#### Participants

In total, 90 Black Americans (*M_age_ =* 35.83, *SD_age_ =*10.34; women = 68.9%) and 93 White Americans (*M_age_ =* 36.66, *SD_age_ =* 9.32; women = 47.3%) were recruited on Amazon Mechanical Turk and paid US$3.

#### Procedure

Participants completed the same reverse-correlation task as in Study 1. In this study, they were informed that they would see a series of images of White Americans and asked to select the individual whom they believed has (a) “mostly African American friends,” (b) “an equal number of White American and African American friends,” or (c) “mostly White American friends.” The base image was the average male Caucasian face from the Karolinska Face Database (see Figure 1; Lundqvist et al., 1998). The same pixel intensity analysis as in Study 1 was conducted with a mask that was adjusted to the base image, resulting in an area of interest of 102,117 pixels.

### Results

The composite classification images for each condition are displayed in the lower half of Figure 2. As in Study 1, an ANOVA revealed that pixel intensities of the images generated by participants differed depending on the friendship condition they had been assigned to, *F*(2, 177) = 12.38, *p* < .001, $\eta_{p}^{2}$ =.19. As illustrated in Figure 3 and Table 1, relative to the White target with mostly White friends, *M =* .54, *SE* = .12, images of the White targets with partly, *M = −*.05, *SE* = .12, and especially mostly Black friends, *M = −*.52, *SE* = .12, were significantly darker. Moreover, the White target with mostly Black friends was significantly darker than the target with partly Black friends. As in Study 1, neither the main effect of the race of participants who generated the images, *F*(1, 177) < .001, *p =* .980, $\eta_{p}^{2}$ < .01, nor its interaction with the friendship manipulation, *F*(2, 175) = 1.45, *p =* .237, $\eta_{p}^{2}$ = .01, were significant.

### Discussion

As predicted, the results from Study 2 mirrored those from Study 1. White targets with primarily White friends were mentally represented as having a lighter skin tone than White targets with networks consisting of mostly or partly Black friends.

## Study 3: The Effect of Friendship Condition on Perceived Prototypicality Controlling for Skin Tone Intensities

Given that phenotypic prototypicality consists of both skin tone and physiognomy (i.e., facial features), it is important to test whether we would observe an effect on people’s perception of Afrocentrism and Eurocentrism controlling for how dark or light the images were. One way to test this would be to present images produced in the first two studies that were photo-edited to be equivalent in pixel intensities to a new sample of participants. However, efforts to produce such images could have an unintended impact by reducing or enhancing contrasts between elements of a face or distorting the image of the face. Therefore, to maintain the actual images generated by the participants in the previous studies, in Study 3, we investigated race-related perceptions of all of the images generated by participants in Studies 1 and 2, controlling for the pixel (skin-tone) intensities of each image.

We recruited a new sample of participants for Study 3, which used a nested design in which ratings (Level 1) were nested within images (Level 2). To be able to adequately test and control for the influence of Level 2 skin tone (pixel intensities), a larger number of images needed to be rated. To provide a sufficient number of images for each person to evaluate, these participants rated either all Black or all White individual classification images generated by the different participants in the reverse-correlation task (not just the final aggregated image) on a dimension of phenotypic prototypicality of primary interest, namely, physiognomy. We expected differences in terms of how African and how European Study 3 participants perceived the physiognomy of each individual to be as a function of believed friendship networks, controlling for pixel intensity in multilevel models.

### Method

#### Participants

A power simulation using SIMR (Green & MacLeod, 2016) indicated that 120 participants would provide more than 90% power to observe small effects (*d =* .30) at the highest level of interaction that the present mixed study design allowed for. Accordingly, 120 White American participants were recruited through Amazon Mechanical Turk (*M_age_* = 41.83, *SD_age_* = 12.35; women = 50.0%) and paid US$3.

#### Procedure

To prevent fatigue, participants were randomly assigned to either rate all 180 images generated of the Black targets in Study 1 or all 183 images generated of the White targets in Study 2. Specifically, participants received a brief explanation of the two dimensions on which race is typically perceived (i.e., physiognomy and skin tone) and were asked to focus on physiognomy and disregard the skin tone when rating the following images. They then rated how African and how European they perceived the physiognomy of each individual to be on 12-point scales (0 *not at all*-11 *very much*). We randomized whether participants first rated all images on the Afrocentrism or the Eurocentrism dimension.

Given the design of the reverse-correlation task in the first two studies, the full factorial design of the present study was 3 (friendship manipulation) × 2 (target race) × 2 (race of image generators). Participants’ ratings (Level 1) were nested within the target images (Level 2) in the data set. Pixel intensity estimates from Study 1 and Study 2 were added as a Level 2 control variable. Because of a high correlation between the target group (White or Black) and the intensity estimates, *r*(22013) = .99, *p* < .001, we standardized the estimate within the Black and White target groups.

### Results

**Figure 4.**
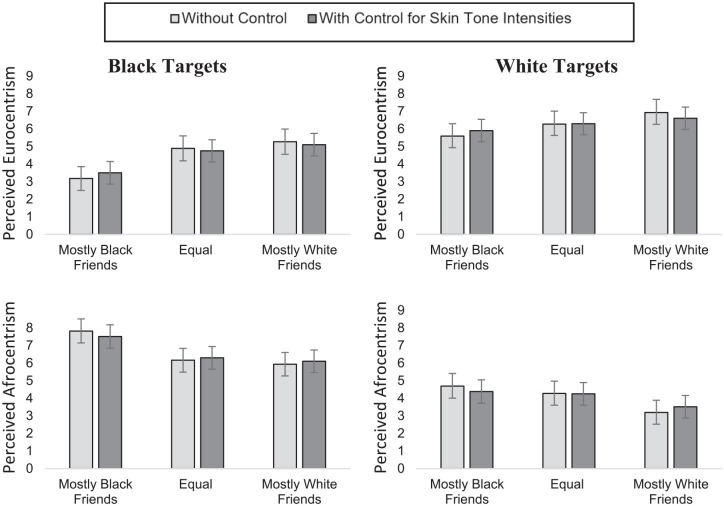
Eurocentrism and Afrocentrism ratings of Black and White targets when controlling versus not controlling for pixel intensities in Study 3. *Note.* Error bars represent 95% confidence intervals.

In each mixed-effects model, intercepts were allowed to vary for participants and targets, and the effect of condition was allowed to vary across participants. We estimated the main effects of the friendship condition, target race, and image generator race as well as the interaction between friendship condition and target race. The three-way interactions did not reach significance in more complex models, *p*s < .287, indicating that image generator race did not moderate the effects. Each model was conducted controlling for pixel intensities. For comparison, we present marginal means with and without this control in Figure 4.

Due to the high number of degrees of freedom that result in very high computing latencies, we report *z* instead of *t*-tests (see Table 1). For Eurocentrism, target group, *F*(1, 149.04) = 36.34, *p* < .001, $\eta_{p}^{2}$ =.20; generator race, *F*(1, 355.06) = 3.89, *p* < .001, $\eta_{p}^{2}$ =.20; the friendship manipulation, *F*(2, 398.91) = 23.80, *p* < .001, $\eta_{p}^{2}$ =.11; and the interaction between target group and the friendship manipulation, *F*(2, 388.96) = 5.09, *p* = .007, $\eta_{p}^{2}$ =.03, reached significance. In addition, pixel intensities positively predicted eurocentrism ratings, *B =* .62, *SE =* .06, *F*(1, 354.97) = 116.00, *p* < .001, $\eta_{p}^{2}$ =.25, As displayed in Figure 4, the effects were quite robust to controlling for these pixel intensities. For Black targets, images generated of targets with mostly Black friends were rated as less Eurocentric than Black targets with an equal number of Black and White friends or mostly White friends. No difference was observed between the images generated of Black targets with mostly White or an equal number of friends from both races. For White targets, images generated of the targets in the mostly White friends condition were rated as more Eurocentric than those generated in the mostly or partly Black friends condition, see Table 1.

For Afrocentrism, target group, *F*(1, 147.34) = 69.40, *p* < .001, $\eta_{p}^{2}$ = .32; the friendship manipulation, *F*(2, 308.24) = 22.76, *p* < .001, $\eta_{p}^{2}$ = .10; and the interaction between target group and the friendship manipulation, *F*(2, 298.29) = 7.03, *p* = .001, $\eta_{p}^{2}$ = .34, reached significance. In addition, pixel intensities negatively predicted Afrocentrism ratings, *B = −*.59, *SE =* .06, *F*(1, 354.91) = 109.71, *p* < .001, $\eta_{p}^{2}$ = .24. The race of the participants who originally generated the face image had no significant effect, *F*(1, 354.94) = 0.01, *p* = .904. $\eta_{p}^{2}$ < .01. For Black targets, images generated in the mostly Black friends condition were rated as more Afrocentric than those generated in the equal number of White and Black friends condition and mostly White friends condition. Ratings of the latter two conditions did not differ significantly. For White targets, images generated in the mostly White friends condition were rated as less Afrocentric than images generated in the equal number of White and Black friends condition, and mostly Black friends condition. No significant difference was observed between the mostly Black friends and an equal number of Black and White friends conditions.

### Discussion

Results from this rating study showed that, although pixel intensities were associated with phenotypic prototypicality ratings, the friendship effects on the latter held when controlling for these pixel intensities. Having established our main predicted phenotypicality effects, in Study 4, we pursued replication of these findings and examined whether the classification images would systematically convey other social information depending on the friendship condition in which the images were generated.

## Study 4: Impressions Associated With the Classification Images on Several Dimensions

In Study 4, an independent sample of participants rated the reverse-correlation composite images from Studies 1 and 2. Our primary aim was to attribute differences in ratings of the classification images, in this case, the group-based aggregated images generated in Studies 1 and 2, to potential biases among the image generators in Studies 1 and 2 by examining the impressions of an independent sample of raters to these images. Yet, we acknowledge that the biases of participants in Studies 1 and 2 that influenced the images they produced may be socially shared biases such that these biases may also attune raters to attach particular, potentially biased, meaning to specific elements of the images generated. Hence, it is not possible to fully disentangle these two influences. For this reason, we included both Black and White raters who were unaware of the previous friendship manipulation. Because there were few effects that were significantly moderated by the race of the raters, we report the results across the rater groups. Further details can be found in the supplementary online materials (SOM).

Study 4 had two aims. The first aim was to test whether perceptions of Afrocentric and Eurocentric appearance of the images would be influenced by the experimental friendship network conditions as in Study 3 but using only the final aggregate images generated in the conditions of Studies 1 and 2 (see Figure 2). Study 4 also assessed a wider range of race-relevant impressions, including perceptions of support for social action for Black or White issues. In Study 4, we focused on responses to the group-based aggregate images (rather than all of the images generated by individual participants, the procedure we used for Study 3, which allowed us to statistically control for pixel intensities) for both methodological and theoretical reasons. Methodologically, because of the greater number of attributes assessed, the number of questions we would pose if we used the full sample of images from the previous studies would be prohibitive (>2,353 questions per participant). Theoretically, the aggregate images represent the shared (pooled) mental representation of a group of African Americans and White Americans believed to have mostly in-group, mostly out-group, or mixed friendship networks. To test the predictions related to the first goal, we examined the two-way interaction between friendship condition and target race while also testing for further moderation by the race of the image generators.

The second aim was to test whether Study 4 participants would rate the images participants in Studies 1 and 2 generated differently depending on whether the targets were racial in- or out-group members to these image generators. Participants rated targets on various dimensions of person perception, including two fundamental dimensions of person and group perception, warmth and competence (Fiske et al., 2002), and key elements of intergroup relations, such as threat, trustworthiness, and status (social class; Dovidio & Gaertner, 2010). The rationale for our hypothesis was that having friends mainly from the target’s own group (e.g., White targets with mainly White friends) or from another group (e.g., White targets with mainly Black friends or an equal number of Black and White friends) may signal very different social orientations depending on whether the respective target is an in- or out-group member. Specifically, we first tested whether *in-group* targets generated by Study 1 and Study 2 participants who believed the targets had mostly same-race friends would be viewed more positively than in-group targets with partly or mostly other-race friends. The rationale for this was that having mostly own-race friends for in-group members may be understood as an act of group solidarity (see Johnson & Ashburn-Nardo, 2014), whereas having partly, and especially mostly, other-race friends may be seen as a betrayal. Second, we tested whether for out-group targets, the opposite may be the case. That is, because it reinforces racial prototypes that define and distinguish different groups (Hogg et al., 2017; Turner et al., 1987), the image of an *out-group* target that was generated based on the belief that the person primarily had same-race friends may convey the impression to participants in Study 4 that the target has a racially exclusionary orientation (Shelton & Richeson, 2005). Such a target would likely produce more negative responses compared to targets with partly or mostly other-race friends, which are the two conditions that make friendships between in-group members and the out-group target salient (Cooley et al., 2018; Wright et al., 1997). Hence, because these effects fundamentally depend on the relationship between the race of the target and the race of the image generators, we calculated Friendship Condition × Target Race × Race of Image Generators interactions. Given the large number of predictors and interactions in this study, we adjusted for multiple tests using the Holm’s *p* correction.

### Method

#### Participants

Because 45 participants per racial group provide 90% power to detect a small effect (*f* = .14/η^2^ = .02; α = .05) in repeated-measurement designs, we recruited 52 Black Americans (*M_age_* = 44.50, *SD_age_* = 16.93; women = 51.9%) and 50 White Americans (*M_age_* = 47.65, *SD_age_* = 16.09; women = 50.9%) using Qualtrics Panels. Both subsamples were demographically, politically, and geographically representative of their populations (see SOM).

#### Procedure

Participants rated the 12 composite images generated in the first two studies (see Figure 2), along with six filler images (two images taken from the stimuli in each study plus both base images) to make the comparisons of interest less salient. Participants first rated, in random order, how (a) Black/Afrocentric, (b) White/Eurocentric, (c) threatening, (d) trustworthy, (e) warm, (f) competent, and (g) lower class the targets looked on 11-point scales (0 *not at all*–10 *very much*). To be consistent with the other items, in the presentation of the findings, the threatening and low-class scores items were reverse-scored such that higher scores represented more favorable perceptions—that is, of being less threatening and of higher social class. Next, in random order, participants rated on 11-point scales (0 *not at all likely*–10 *very likely*) how likely they believed that the target individuals would (a) volunteer to work with drug addicts in a predominantly Black community, (b) join a Black Lives Matter march, (d) volunteer to work with drug addicts in a predominantly White community, and (d) join a march for keeping Confederate monuments. To keep the presentation simple, the two items measuring support for Black issues, *r*(1298) = .50, *p* < .001, and two items measuring support for White issues, *r*(1299) = .35, *p* < .001, were each combined into one race-based social action score. Results for the individual items and three additional items that corresponded to the general pattern of results can be found in the SOM.

### Results

#### Euro- and Afrocentrism

The first goal of Study 4 was to test whether subjective perceptions of Eurocentric and Afrocentric appearance of the aggregated classification images of the Black and White targets would be influenced by the experimental friendship conditions under which participants in Studies 1 and 2 generated the images. Because we used the final aggregated images as stimuli rather than the full set of all of the individual images generated, the analyses do not control for pixel intensity. That is, because pixel intensities are a Level 2 variable, such analyses would be severely underpowered given the present design. Full model details can be found in the SOM, including analyses testing differences between Black and White raters. The predicted Friendship Manipulation × Target Race interactions were significant for Eurocentrism, *F*(2, 961) = 452.69, *p* < .001, $\eta_{p}^{2}$ =.45, as well as for Afrocentrism, *F*(2, 962) = 395.27, *p* < .001, $\eta_{p}^{2}$ =.42. Black targets believed to have mostly Black friends were rated as less Eurocentric, *M* = 0.82, *SE =* .17, and more Afrocentric, *M* = 8.70, *SE =* .16, than Black targets generated in the equal Black and White friends condition (Eurocentrism: *M* = 3.77, *SE=*.24, Afrocentrism: *M* = 5.04, *SE =* .16) and Black targets generated in the mostly White friends condition (Eurocentrism: *M* = 3.84, *SE =* .25, Afrocentrism: *M* = 5.23, *SE=*.27; see Table 1 for contrasts). White targets believed to have mostly White friends were rated as more Eurocentric, *M* = 7.96, *SE =* .18, and less Afrocentric, *M* = 1.27, *SE =* .17, than White targets generated in the equal Black and White friends condition (Eurocentrism: *M* = 4.84, *SE =* .21, Afrocentrism: *M* = 3.48, *SE =* .23) and White targets generated in the mostly Black friends condition (Eurocentrism: *M* = 3.46, *SE =* .21, Afrocentrism: *M* = 4.34, *SE =* .24). These findings replicated the results of Study 3.

**Figure 5.**
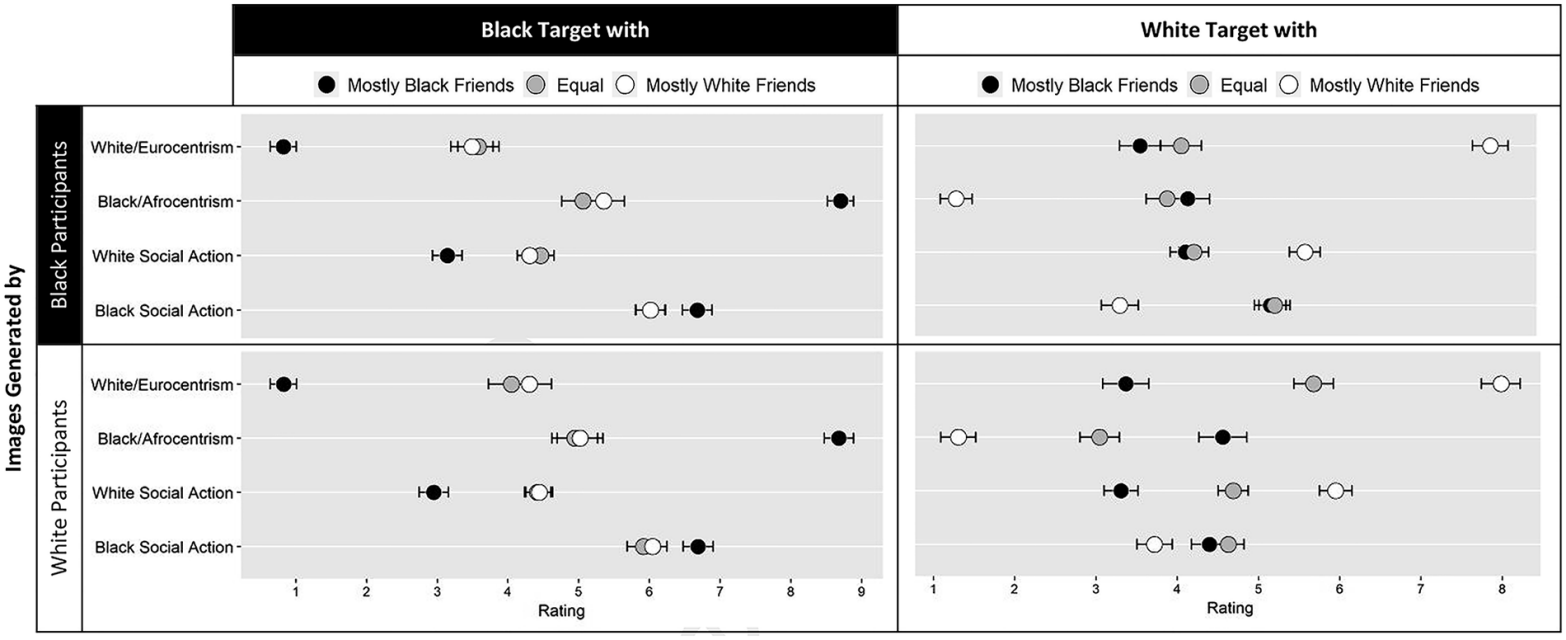
Phenotypicality and social action ratings by the independent samples of raters in Study 4 are presented for classification images generated in the different friendship conditions by Black and White participants. *Note.* Error bars represent ±1 standard error.

Having established these effects, we ran further analyses to explore the role of the image generators’ race. Significant but weak three-way interactions between the friendship manipulation, target race, and race of the image generators were observed for Eurocentrism ratings, *F*(2, 961)= 8.44, *p* = .002, $\eta_{p}^{2}$ =.02, and Afrocentrism ratings, *F*(2, 962) = 3.90, *p* = .016, $\eta_{p}^{2}$ =.01. As can be seen in Figure 5, when the target in the reverse-correlation study was Black, the effect of the friendship network on the images generated by both Black and White participants and the ratings were comparable. However, when the target was White and images were generated by White participants, the image of the White target with an equal number of White and Black friends was rated in between the target with mostly White friends and the target with mostly Black friends (see Figure 5). This was not the case for images of White targets generated by Black participants for which the main difference was between White targets with mostly White friends and targets with an equal number of White and Black friends or mostly Black friends.

#### Social action for White and Black issues

As with the ratings of Afrocentric and Eurocentric appearance, a significant Friendship Manipulation × Race of the Target interaction effect was observed for White social action, *F*(2, 962) = 206.50, *p* < .001, $\eta_{p}^{2}$ = .28, as well as Black social action, *F*(2, 963) = 87.77, *p* < .001, $\eta_{p}^{2}$ = .12. Black targets believed to have mostly Black friends were rated as less likely to support White social action, *M* = 3.05, *SE =* .16, and more likely to support Black social action, *M* = 6.70, *SE =* .17, than Black targets generated in the equal Black and White friends condition (White social action: *M* = 4.44, *SE=*.19, Black social action: *M* = 6.00, *SE =* .22) and Black targets generated in the mostly White friends condition (White social action: *M* = 4.37, *SE =* .19, Black social action: *M* = 6.06, *SE =* .20; see Table 1). No differences were observed between the Black targets with partly or mostly White friends.

White targets believed to have mostly White friends were rated as more likely to support White social action, *M* = 5.78, *SE =* .16, and less likely to support Black social action, *M* = 3.49, *SE =* .18, than White targets generated in the equal Black and White friends condition (White social action: *M* = 4.46, *SE =* .16, Black social action: *M* = 4.93, *SE =* .18) and White targets generated in the mostly Black friends condition (White social action: *M* = 3.71, *SE =* .16, Black social action: *M* = 4.78, *SE =* .17).

Having established the effects most relevant to our first goal, we tested whether the observed effects would be further moderated by the race of the participants generating the images. The three-way interactions between friendship condition, target race, and race of the image generators were significant but weak for White social action ratings, *F*(2, 962) = 10.98, *p* < .001, $\eta_{p}^{2}$ = .02, and Black social action ratings, *F*(2, 963) = 6.83, *p* = .014, $\eta_{p}^{2}$ = .01. The pattern represented by the three-way interactions for White social action and for Black social action mapped directly onto the results for Eurocentrism and Afrocentrism, described in the previous section (see Figure 5).

Additional analyses showed that the effects on social action ratings were partly mediated by Afrocentrism and Eurocentrism ratings (see SOM). These analyses showed that one reason why images of targets with other-race friends were seen as more likely to support the racial out-group was that they were perceived to be more racially similar to them.

#### Traits

**Figure 6.**
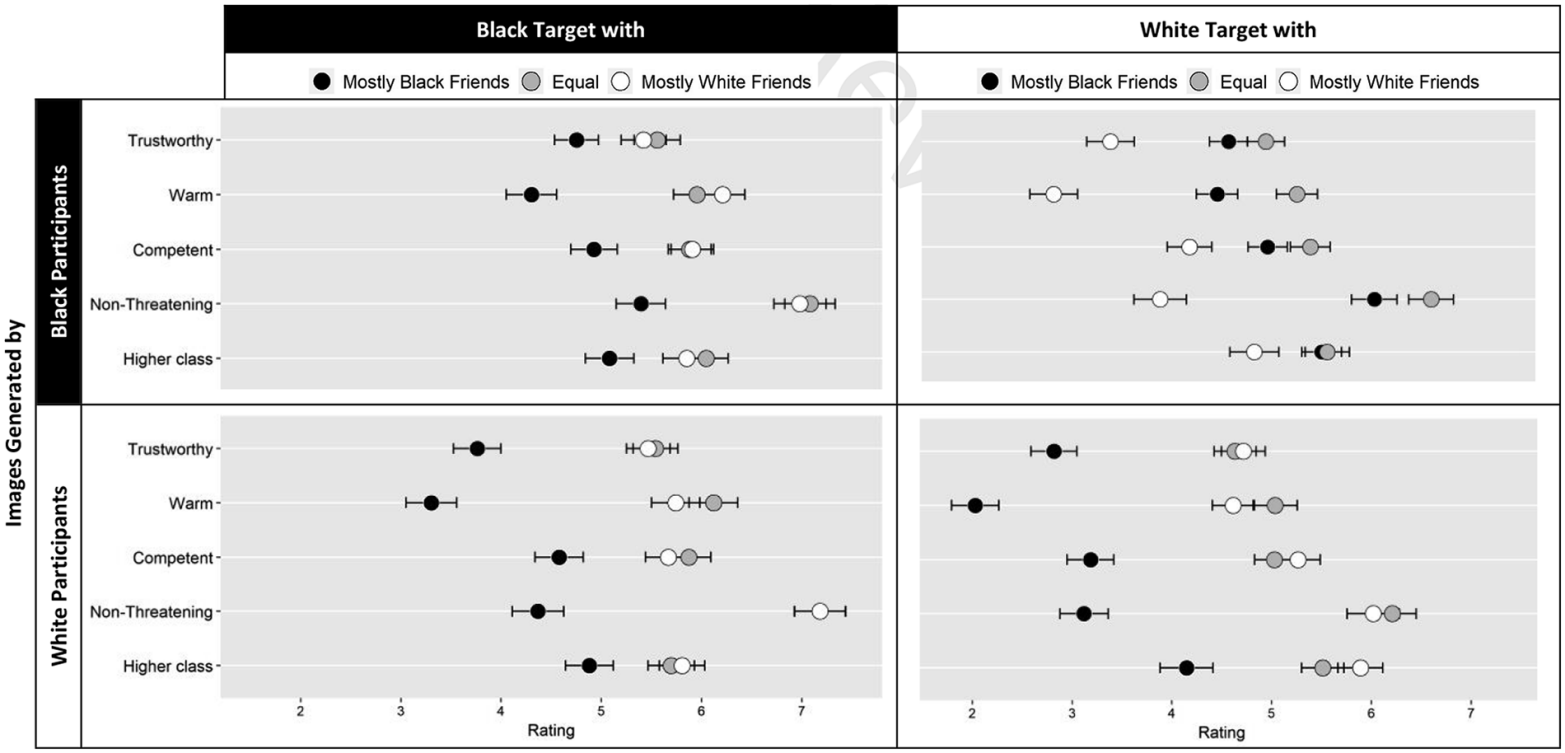
Trait ratings by the independent samples in Study 4 are presented for classification images generated in the different friendship conditions by Black and White participants. *Note*. Error bars represent ±1 standard error.

With respect to our second goal, we tested whether (a) images of in-group members with mostly same race friends would be rated more positively than images of in-group members with partly and, especially, mostly other-race friends, and (b) out-group members with mostly own-race friends would be rated more negatively than out-group members with partly or mostly other-race friends. Indeed, the three-way interaction was significant for each trait, trustworthy: *F*(2, 963) = 58.87, *p* < .001, $\eta_{p}^{2}$ = .10; warmth: *F*(2, 963) = 61.16, *p* < .001, $\eta_{p}^{2}$ = .10; competence: *F*(2, 963) = 31.88, *p* < .001, $\eta_{p}^{2}$ =.06; nonthreatening: *F*(2, 962) = 87.86, *p* < .001, $\eta_{p}^{2}$ =.14; higher social class: *F*(2, 961) = 24.64, *p* < .001, $\eta_{p}^{2}$ = .05. The means for each condition and each measure are displayed in Figure 6, and contrasts between the conditions in Table 2.

**Table 2.** Contrast for Trait Ratings in Study 4 of Images That Were Generated by Black and White Participants in Studies 1 and 2.

Study / Variable	Mostly own-race vs. equal number of own and other-race				Mostly own-race vs. mostly other-race				Equal number of own and other- race vs. mostly other-race			
	*t*	*df*	*p*	*d_rm_*	*t*	*df*	*p*	*d_rm_*	*t*	*df*	*p*	*d_rm_*
Black Target												
Black image generators												
Trust	−3.52	424	<.001	0.56	−3.11	530	.002	0.47	0.70	714	.484	0.10
Warmth	−6.05	365	<.001	1.01	−7.67	490	<.001	1.48	−1.16	700	.247	0.16
Competence	−4.34	480	<.001	0.67	−4.71	578	<.001	0.69	−0.16	727	.875	0.02
Threat	5.99	446	<.001	0.95	6.10	599	<.001	0.88	−0.46	682	.646	0.06
Social Class	4.53	466	<.001	0.70	3.70	506	<.001	0.56	−1.04	741	.300	0.14
White image generators												
Trust	−7.71	424	<.001	1.23	−7.92	530	<.001	1.19	0.32	714	.747	0.04
Warmth	−10.23	365	<.001	1.71	−9.71	490	<.001	1.49	1.65	700	.099	0.23
Competence	−5.95	480	<.001	0.92	−5.30	578	<.001	0.78	1.02	727	.307	0.14
Threat	10.11	443	<.001	1.58	10.89	599	<.001	1.57	−0.01	680	.992	<0.01
Social Class	3.76	466	<.001	0.58	4.40	506	<.001	0.67	0.62	741	.537	0.08
White target												
Black image generators												
Trust	−7.14	424	<.001	1.14	−5.78	530	<.001	0.87	1.99	714	.047	0.27
Warmth	−9.28	365	<.001	1.55	−6.82	490	<.001	1.04	3.69	700	<.001	0.51
Competence	−5.75	480	<.001	0.88	−3.99	578	<.001	0.58	2.20	727	.028	0.30
Threat	10.03	446	<.001	1.58	8.71	602	<.001	1.26	−2.30	680	.022	0.32
Social Class	3.59	469	<.001	0.56	3.33	509	<.001	0.51	−0.37	743	.712	0.05
White image generators												
Trust	0.33	424	.743	0.05	8.84	530	<.001	1.32	9.23	714	<.001	1.27
Warmth	−1.59	365	.113	0.27	10.35	490	<.001	1.58	13.36	700	<.001	1.85
Competence	1.11	480	.266	0.17	10.13	578	<.001	1.48	9.56	727	<.001	1.31
Threat	0.72	443	.470	0.11	−11.23	599	<.001	1.62	−12.45	680	<.001	1.74
Social Class	−1.83	466	.068	0.28	−8.42	506	<.001	1.28	−7.30	741	<.001	0.99

We used analytic contrasts to test the Study 4 predictions. Study 4 participants had different impressions of the images that participants in Studies 1 and 2 generated of in-group members depending on their friendship circles. For images that White participants generated of White targets (i.e., their in-group members), targets with mostly or partly own-race friends were consistently rated more positively than the target with mostly out-group friends. By contrast, for the images that Black participants generated of Black targets (i.e., their in-group members), targets with mostly own-race friends were consistently rated more negatively than targets with partly or mostly other-race friends, although the difference for Black participants was not as stark as that for White participants (see Figure 6 and Table 2).

The results further demonstrated that out-group members with mostly own-race friends were rated more negatively than out-group members with mostly or partly other-race friends. For images that White participants in Study 1 generated of Black targets (i.e., their out-group members), the target with mostly own-race friends was consistently rated more negatively than the targets with mostly or partly other-race friends (in Figure 6 and Table 2). For images that Black participants in Study 2 generated of the White targets, the same pattern was observed. Targets with mostly own-race friends were rated more negatively than targets with partly and, especially, mostly other-race friends.

Additional analyses presented in SOM show that the effect of the friendship condition on the trait ratings remained significant when controlling for Afrocentrism and Eurocentrism.

### Discussion

In terms of the first goal of this study, we replicated the effects of friendship networks on the race-related appearance of Black and White targets observed in our previous studies. That is, findings converged with those from pixel intensity tests in Studies 1 and 2 and the ratings of individual images from Study 3. Specifically, Study 4 participants rated the appearance of aggregated images of Black and White targets generated in the mostly own-race friends conditions as more phenotypically prototypical of their respective racial in-groups than the images of targets with partly or mostly other-race friends. For both target groups, participants further rated the images of targets generated in the mostly own-race friends condition to be most supportive of social action benefiting their in-group and least supportive for social action benefiting the respective out-group.

With respect to the second goal of this study, we extended our three previous investigations by demonstrating that the impact of friendship networks on impressions systematically differed when the target and the image-generating participant (in Studies 1 and 2) were of the same race compared with when they were of different races. Significant three-way interactions involving the friendship condition, the target race, and the race of image generators in Studies 1 and 2 emerged for all trait measures. When it came to our first prediction, that images of in-group targets with mostly own-race friends would be evaluated more positively than those with mostly or partly other-race friends, results were mixed. Images generated by White participants of racial in-group members with mostly own-race friends or an equal number of own- and other-race friends were evaluated more positively than the target generated in the mostly other-race condition. However, for images generated by Black participants, images of racial in-group members with mostly own-race friends were evaluated more negatively than images of in-group members with mostly and partly other-race friends. Hence, the White generators seemed to devalue and potentially socially sanction racial in-group members who show strong social affiliation with the out-group, whereas Black image generators seemed to have a positive stance toward in-group members with other-race friends. This difference may make sense from a status perspective. Because high-status groups often are invested in maintaining the hierarchical status quo (Sidanius & Pratto, 1999), it is possible that White Americans would have especially negative representations of White targets who have a substantial number of Black friends because this type of friendship network may signal betrayal of the dominant group and weakening of its dominant position. By contrast, Black Americans might either feel positively toward Black targets with many White friends or perhaps believe that Black targets with many White friends must appear very friendly to be accepted by White people.

Concerning the ratings of images of out-group members, findings for Black and White image generators converged. In both groups, we found support for the prediction that out-group targets with mostly own-race friends would be evaluated more negatively than those with partly or mostly other-race friends. Arguably, the image generators in Studies 1 and 2 thought that out-group targets with mostly own-race friends have exclusionary intergroup orientations, which generally tend to be evaluated negatively, whereas they thought that out-group targets with other-race friends have inclusionary orientations, which typically are viewed positively (Shelton & Richeson, 2005; Wout et al., 2010).

Additional analyses presented in SOM showed that the observed effects of friendship manipulation on perceived social action tendencies were partly, but not fully, explained by changes in phenotypic prototypicality. This finding suggests that changes in the target’s face beyond phenotypic prototypicality may have contributed to the effects of friendship network on perceived social action tendencies. For instance, a visual inspection of the images shows that some of the depicted individuals have slight smiles, which arguably may convey more open-mindedness and tolerance, thereby influencing the ratings of these individuals.

## General Discussion

Although rare in many spheres of life in the United States (e.g., in residences and schools), racial integration is generally viewed positively (Pew Research Center, 2017) and supported formally by legal interventions of the United States Supreme Court. Moreover, having out-group friends robustly predicts positive attitudes toward the out-group as a whole (Davies et al., 2011), and even simply becoming aware that an in-group member has out-group friends (i.e., extended contact) can improve one’s intergroup attitudes (Turner et al., 2008; Wright et al., 1997; Zhou et al., 2019). However, to the extent that the out-group is generally perceived less favorably than the in-group, having a substantial number of other-race friends may be stigmatizing for an individual and thus have a social cost (Hernandez et al., 2016). In four studies with samples of Black and White Americans, the present research demonstrated the pervasive effects of interracial friendships on perceptions of race, group solidarity, and major trait dimensions. Across the race of the targets and the race of participants who generated the images, target individuals whose friends partly or mostly belonged to the racial out-group were mentally represented as phenotypically more similar to the racial out-group compared to individuals who had mostly same-race friends. This racial assimilation effect was cross-validated by pixel intensity analyses (Studies 1 and 2) and by independent raters who were unaware of the hypotheses (Studies 3 and 4).

Our results highlight social perceptual processes through which interracial friendships may improve intergroup relations. The images of individuals with racial out-group friends were judged as more supportive of the social causes of their friends’ racial group. Moreover, both Black and White participants positively represented racial out-group members who had other-race friends. Hence, by producing positive representations that look phenotypically similar to the racial out-group, interracial friendships may lead to perceptions of shared group membership and interracial solidarity. At a social perceptual level, this process may explain why contact often leads to common group identifications and why the mere third-person observation of positive intergroup contact often is sufficient to reduce prejudice (Christ et al., 2014; Turner et al., 2008; Wright et al., 1997).

However, our findings also indicate that mental representations resulting from extended contact can have negative and unintended consequences for intergroup relations. Black and White participants alike had the most negative representations of racial out-group members whose friends mostly belonged to the same race, which is the social default in the United States. White participants did not show this derogatory tendency toward members of their own racial group whose friends mostly belonged to the same race. Such a finding is consistent with social dominance theory (Sidanius & Pratto, 1999), which argues that the observed asymmetry is more typical for White participants (members of the socially dominant racial group in the United States) who want to defend their dominant position than for Black participants (a nondominant racial group). Indeed, in contrast to Johnson and Ashburn-Nardo (2014), who found that Black participants responded less positively (with less empathy) to other Blacks with close White friends, our results revealed that Black participants had generally positive representations of in-group members with White friends. It is possible that the nature of the context in which participants were asked to make judgments may moderate these different effects. Participants in the present research offered initial impressions of others based merely on their pattern of in-group and out-group friendships. Johnson and Ashburn-Nardo (2014) investigated Black participants’ empathic responses to another Black person in need (because of the loss of parents in a car accident). The authors suggested that the reason why Black participants had less empathy for Blacks with close White friends was because the person violated the “Black code” of racial solidarity. Thus, it is possible that the situation featuring the need for support employed by Johnson and Ashburn-Nardo made the value of Black racial solidarity particularly salient, in a way that our situation did not.

## Constraints on Generality

Our findings replicated across different studies and samples of Black and White Americans, mostly recruited through online panel platforms. Given the consistency of findings and the size of effects, across studies, including in Study 4 that used a representative sample, we would expect the results to replicate also among samples of Black and White Americans obtained through different means. Yet, the extent to which our findings relating to the effects of friendship networks generalize to relations between other racial or ethnic groups in the United States or to Black, White, or other groups in other countries remains uncertain. Because the positive effects of intergroup friendships on intergroup attitudes occur robustly for relations between different types of groups and across international contexts (Cooley et al., 2018; Turner et al., 2008; Wright et al., 1997; Zhou et al., 2019), the effects of friendship networks may apply to a broad range of groups and settings (e.g., Asian Americans with different friendship networks). However, because of its unique history (involving slavery and its legacy), Black–White relations in the United States might be sufficiently distinctive that generalizability is limited. Relatedly, we note that our interracial friendship conditions assessed mental representations of targets with friendship networks that are quite unusual in the United States: It is very rare for Black Americans, and especially for White Americans, to have an equal number of friends from both groups and, even more rare, to have mostly friends from the respective racial out-group (Cox & Jones, 2016). Because having half or more of one’s friends being from a different racial group is so rare socially among Black and White Americans, and non-normative behavior elicits strong dispositional attributions, the distinctiveness of this information may account for the relatively potent and robust effects we observed across our studies. Thus, it is possible that the effects of networks containing a substantial number of out-group friends on mental representation may be stronger in cultures in which the relevant groups are more residentially segregated. Another implication is that for some types of groups, such as gender groups for which intergroup friendships are common and highly normative, the effects would be weaker.

Our work reveals systematic effects of beliefs about how a person’s friendship network affects the ways in which that individual is mentally represented (in terms of skin tone and facial appearance). Nevertheless, we acknowledge that the current work cannot definitively identify the specific process determining this effect. Multiple explanations remain possible. For example, we based our hypothesis on the assumption that people would generate images influenced by beliefs about the target’s friendship preferences. Yet, it is possible that image generators were focusing on the assumed preferences of members’ different racial groups for the kinds of targets that they would accept as friends. Participants who generated images of White targets might have assumed that the racial out-group would be more accepting of someone with a more similar skin tone or phenotypic appearance to them and thus generated a darker-skinned White target with more Afrocentric features. Illuminating the degree to which perceptions of the target preferences and assumptions about potential friends’ preferences underlie the way images are generated has valuable theoretical and practical implications and thus represents an important avenue for future research. Understanding the influences that represent the basis of the effects we observed can offer new perspectives on the dynamics and consequences of, for example, extended intergroup contact as well as highlighting more generally the complex dynamics of intergroup exchanges.

We focused on the effects of information about friendship networks because previous research has identified that intergroup friendship is a particularly potent factor in shaping intergroup relations (Davies et al., 2011; Paolini et al., 2021; Wright et al., 1997). However, we acknowledge that other kinds of information may have similar effects. For example, in previous research, minority-group members who adopted the U.S. culture were represented as phenotypically more White than those separating from it (Kunst, Dovidio, & Dotsch, 2018). Thus, any type of behavior that signals affiliation with the out-group, be it in terms of food, clothing, cultural styles, or friendships, may produce similar effects. It is possible, however, that some aspects may play particularly influential roles. For instance, both intergroup friendship and the adoption of culture reflect intentions expressed at an individual and a societal level for intergroup integration and harmony. If intentional commitment represents a critical factor, then seemingly harmonious activities such as appearing in close proximity or having common but superficial interests (e.g., in television shows) would likely have a weaker effect and possibly not have sufficient impact to produce different mental images of members of the different groups. However, there is work that finds Whites with stereotypically Black interests are also represented as more Black, indicating that shared interests might indeed be enough to trigger this effect (Hinzman & Maddox, 2017). In this respect, we acknowledge that the design of our studies leaves room for the alternative explanation that when reading a certain description that is more typical for one group than the other, participants may pick the individual who phenotypically is most closely associated with the characteristic or activity. From this perspective, having friends from another group may be just one instantiation of a characteristic of this type rather than being a distinctively influential characteristic.

The main goals of the current research were to determine whether target race and the racial composition of friendship networks affect the mental representations that people have of targets and to determine whether these images alone would convey impressions in systematic ways to others. Our results demonstrated the predicted differences in racial prototypicality and in general dimensions of personal evaluation. However, the images generated by participants in Studies 1 and 2 are complex stimuli that vary in multiple ways, such as skin tone, race-related facial features, and facial expressions. Moreover, they look relatively artificial compared to real photos. Building on our initial findings, future work might consider more fully how the facial images vary, including in terms of the appearance of the eyes, nose, mouth, skin tone, and emotional expressions and then map, by quantifying the specific feature-to-impression relationships, these differences onto particular aspects of the impressions (e.g., warmth and competence) that independent observers form. For instance, an investigation could directly explore how facial expression varies as a function of target race and friendship network, and then how those facial expressions affect ratings of warmth and competence, or whether the patterns described here are robust when directly accounting for the target affect. Such research might also use stimuli that are more realistic than the images commonly produced and presented in reverse-correlation research. For example, researchers may employ techniques that allow them to independently alter the skin tone and physiognomy of the images without compromising the realism of these images to create more ecologically valid stimuli.

## Conclusion

Having mostly same-race friends is the social default in the United States. In the present research, we demonstrated how mental representations of individuals’ race and traits may change once their friendship circles are believed to diverge from this default. Target individuals believed to have partly and mostly racial out-group friends were mentally represented as having a racial appearance similar to that of their friends and were believed to more strongly support issues benefiting the members of the other racial group. Despite being the social default, participants also tended to have negative mental representations of racial out-group members who affiliated mostly with same-race individuals, potentially because it reflects a racially exclusionary orientation. Hence, the present research, theoretically, offers new insights into how information about the racial composition of others’ friendship patterns can shape impressions and expectations of in-group and out-group members that, practically, can have immediate and important social implications for intergroup relations.

## Supplemental Material

sj-docx-1-psp-10.1177_01461672211024118 – Supplemental material for Knowledge About Individuals’ Interracial Friendships Is Systematically Associated With Mental Representations of Race, Traits, and Group Solidarity Click here for additional data file. Supplemental material, sj-docx-1-psp-10.1177_01461672211024118 for Knowledge About Individuals’ Interracial Friendships Is Systematically Associated With Mental Representations of Race, Traits, and Group Solidarity by Jonas R. Kunst, Ivuoma N. Onyeador and John F. Dovidio in Personality and Social Psychology Bulletin
