# Supplementary material for: Knowledge About Individuals’ Interracial Friendships Is Systematically Associated With Mental Representations of Race, Traits, and Group Solidarity
Source: Pers Soc Psychol Bull. 2021 Jun 19;48(5):718–34. doi: 10.1177/01461672211024118 (PMC9066664; doi:10.1177/01461672211024118)

**Studies 1 & 2**

**[Instruction before trials start]**

**Instructions**

In the following task, we will present pairs of different looking [Study 1: Black; Study 2: White] American faces.

**In each trial, we would like you to select the individual who looks like he has [depending on condition: mostly African Americans friends / mostly White American friends / an equal number of White and African American friends]**
 
The task will take some time and can be repetitive. It is nevertheless **very important** that you concentrate and have full attention throughout the task. Thank you.

Please click below to confirm that you have understood these instructions.

[ ] Yes, I understand the instructions.

**[Example Trial Study 1]**

Please select the individual who looks like he has [depending on condition: mostly African Americans friends / mostly White American friends / an equal number of White and African American friends]


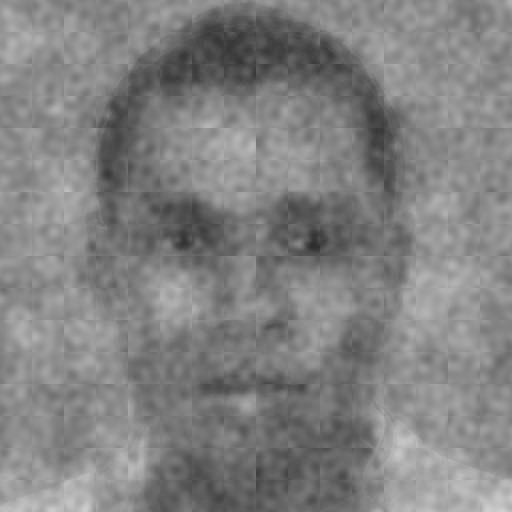

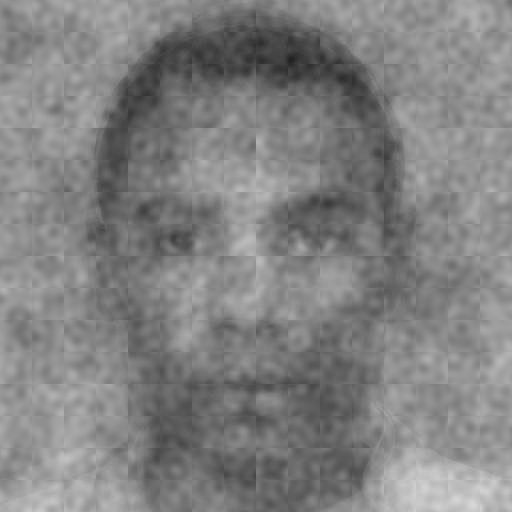


**[Example Trial Study 2]**

Please select the individual who looks like he has [depending on condition: mostly African Americans friends / mostly White American friends / an equal number of White and African American friends]

**
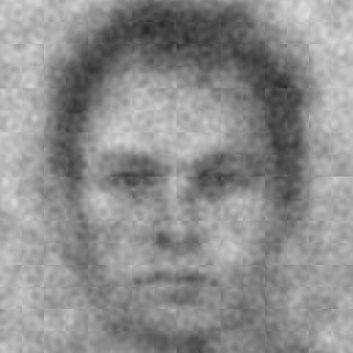

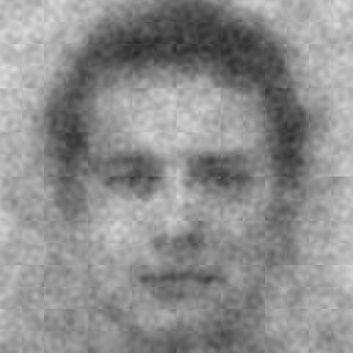
**

**Study 3 (ratings)**

People vary in how typical of their racial group they look. Two dimensions that researchers commonly distinguish between are physiognomy and skin tone.

**Physiognomy**deals with facial features such as a persons' nose, lips, eyes, eyebrows or facial shape.

**Skin tone**simply deals with how light or dark a person's skin is.

In the next task, we will show you a series of individuals, and for each ask you how **Afrocentric (typical for African Americans)**their physiognomy is. Please ignore the skin tone of the targets in this task and solely focus on the individuals' physiognomy, that is, their facial features.

[image placeholder]

How typically **African** do you think the facial physiognomy of the individual is?


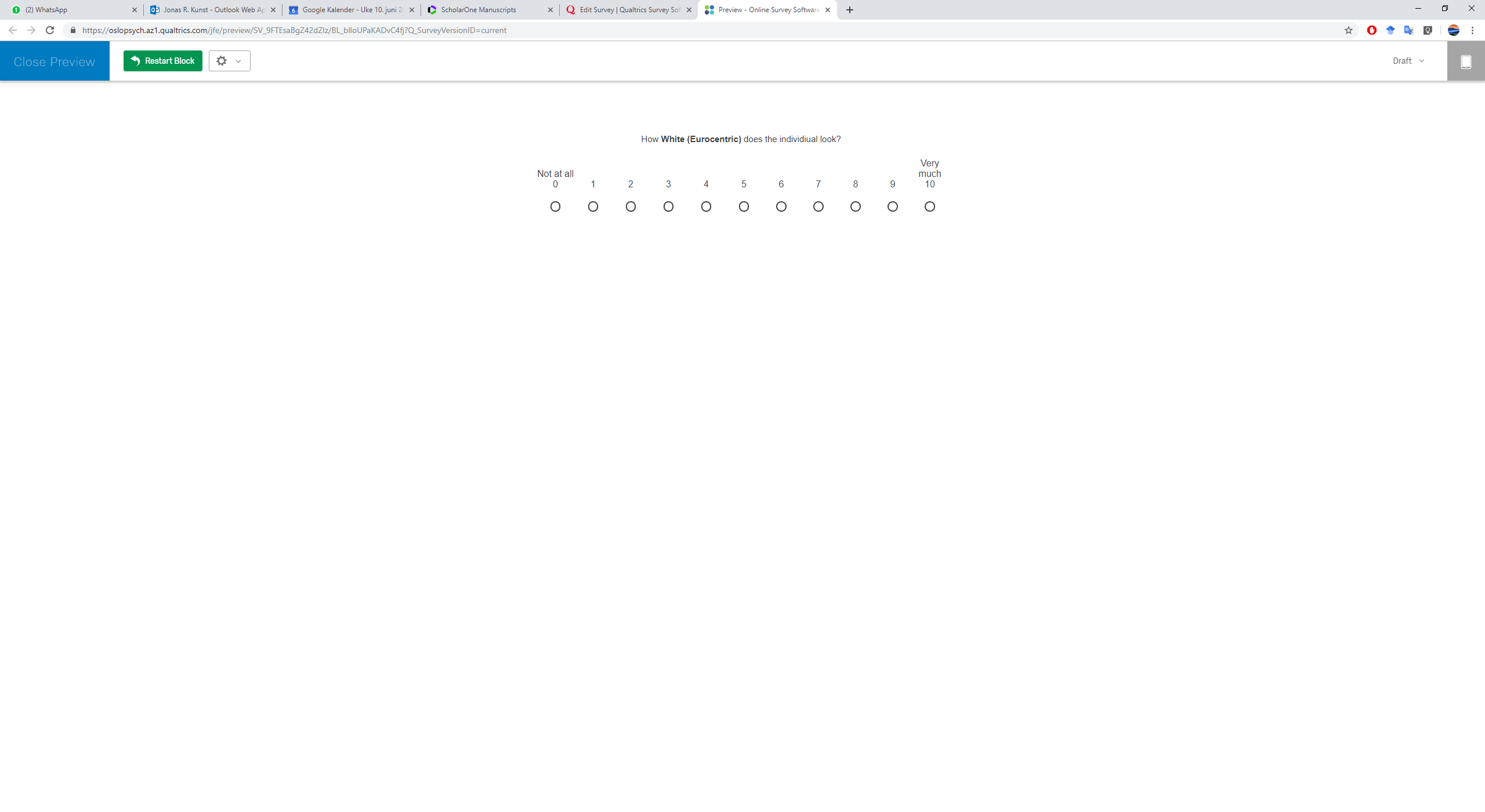


People vary in how typical of their racial group they look. Two dimensions that researchers commonly distinguish between are physiognomy and skin tone.

**Physiognomy**deals with facial features such as a persons' nose, lips, eyes, eyebrows or facial shape.

**Skin tone**simply deals with how light or dark a person's skin is.

In the next task, we will show you a series of individuals, and for each ask you how **Eurocentric (typical for European Americans)**their physiognomy is. Please ignore the skin tone of the targets in this task and solely focus on the individuals' physiognomy, that is, their facial features.

[image placeholder]

How typically **European** do you think the facial physiognomy of the individual is?


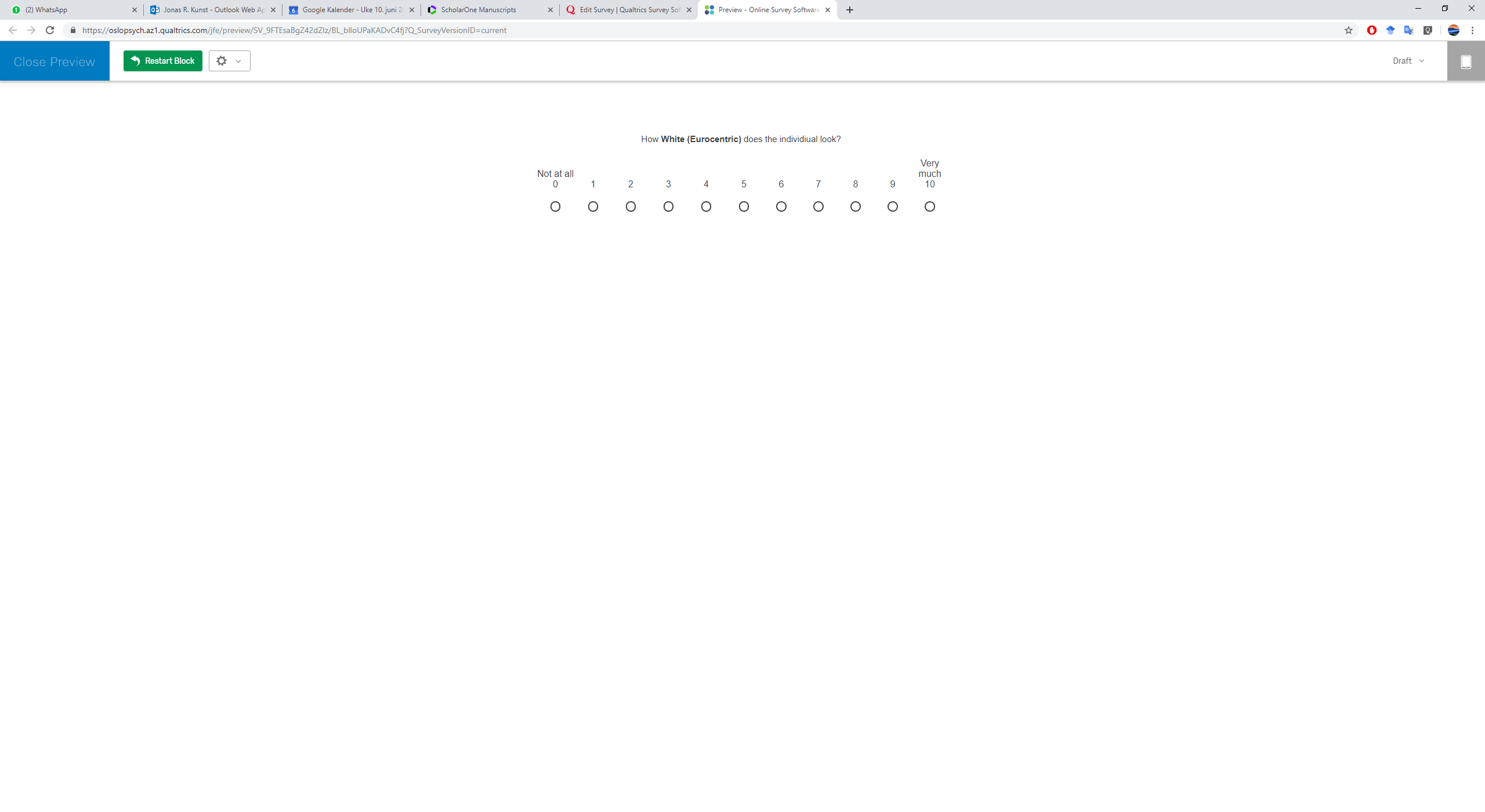


**Study 4 (ratings)**

**Instructions**

In the following task, we will ask you to rate different looking faces. The images will be noisy. It is important that you ignore the noise and focus on the faces.

Please click below to confirm that you have understood these instructions.

[ ] Yes, I understand the instructions.

[The following questions were asked for each of the composite images and filler items using a loop. The presentation order of images and measures was randomized.]

**[First set of measures]**

**[Eurocentrism measure]**

**Please note:**
We will now ask you to rate how White (Eurocentric) the individuals look.

[page break]

[image placeholder]

How **White (Eurocentric)** does the individual look?


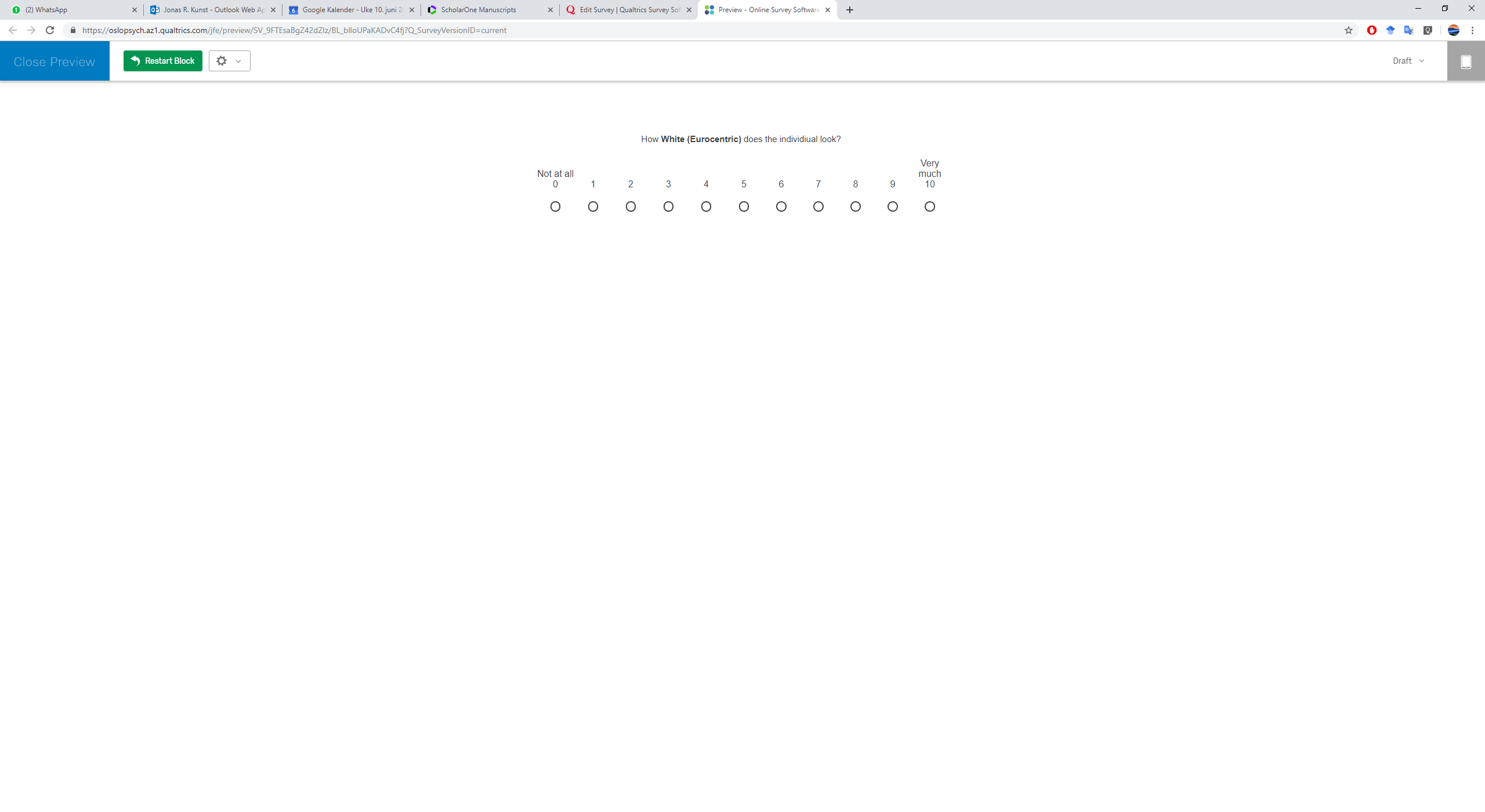


**[Afrocentrism measure]**

**Please note:**
We will now ask you to rate how Black (Afrocentric) the individuals look.

[page break]

[image placeholder]

How **Black (Afrocentric)** does the individual look?


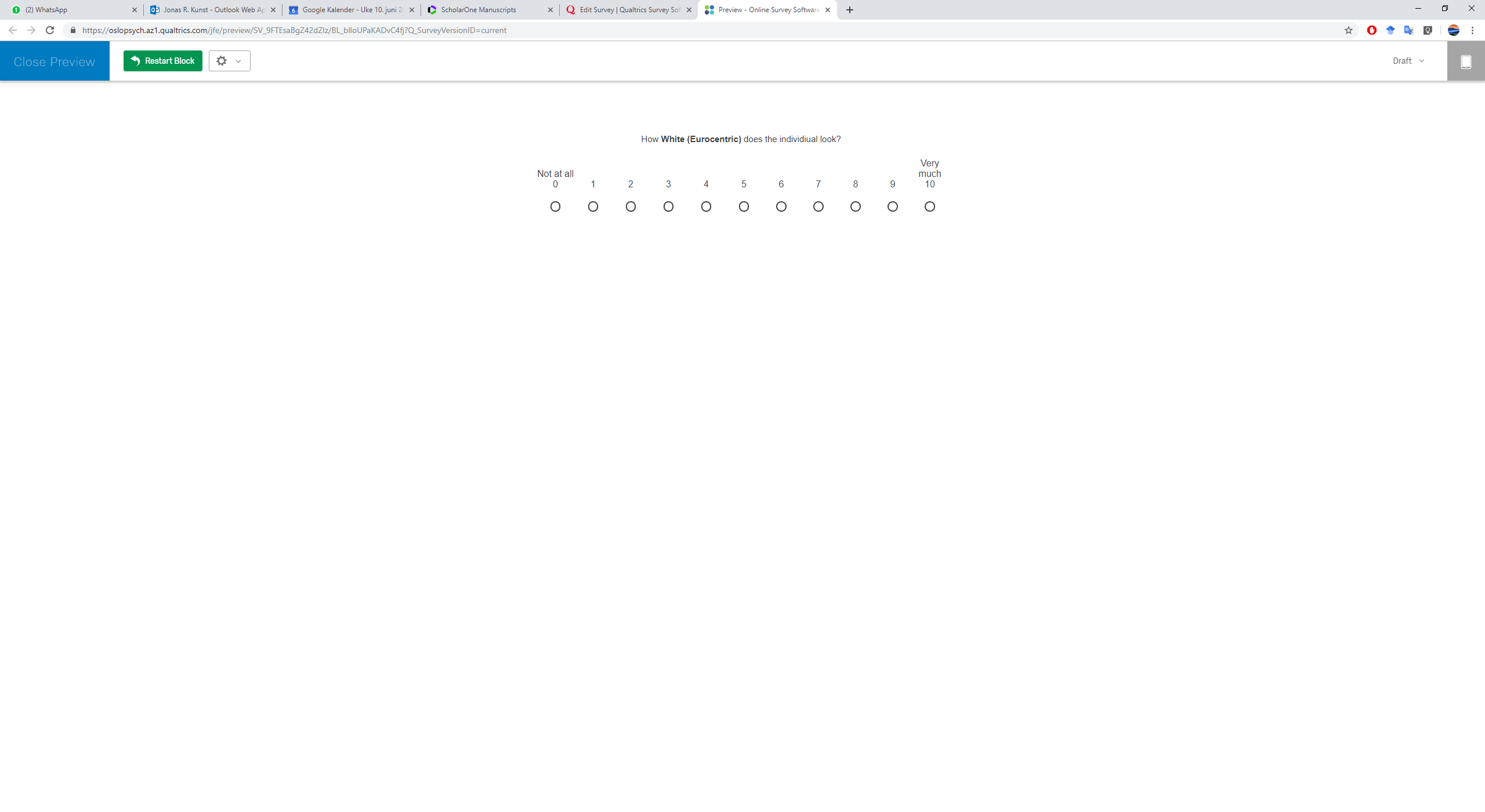


**[Trustworthiness measure]**

**Please note:**
We will now ask you to rate how trustworthy the individuals look.

[page break]

[image placeholder]

How **trustworthy** does the individual look?


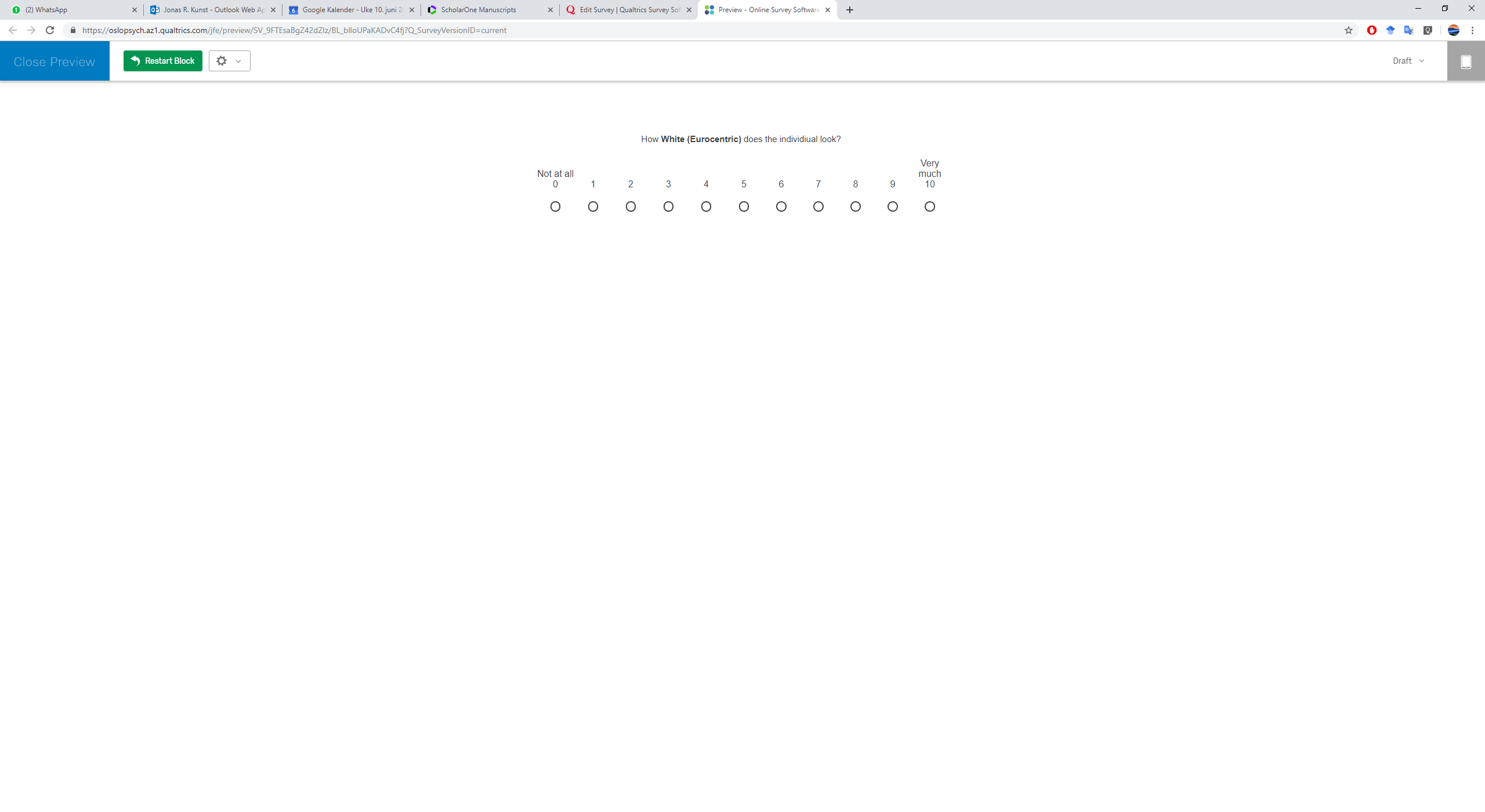


**[Threat measure]**

**Please note:**
We will now ask you to rate how threatening the individuals look.

[page break]

[image placeholder]

How **threatening** does the individual look?


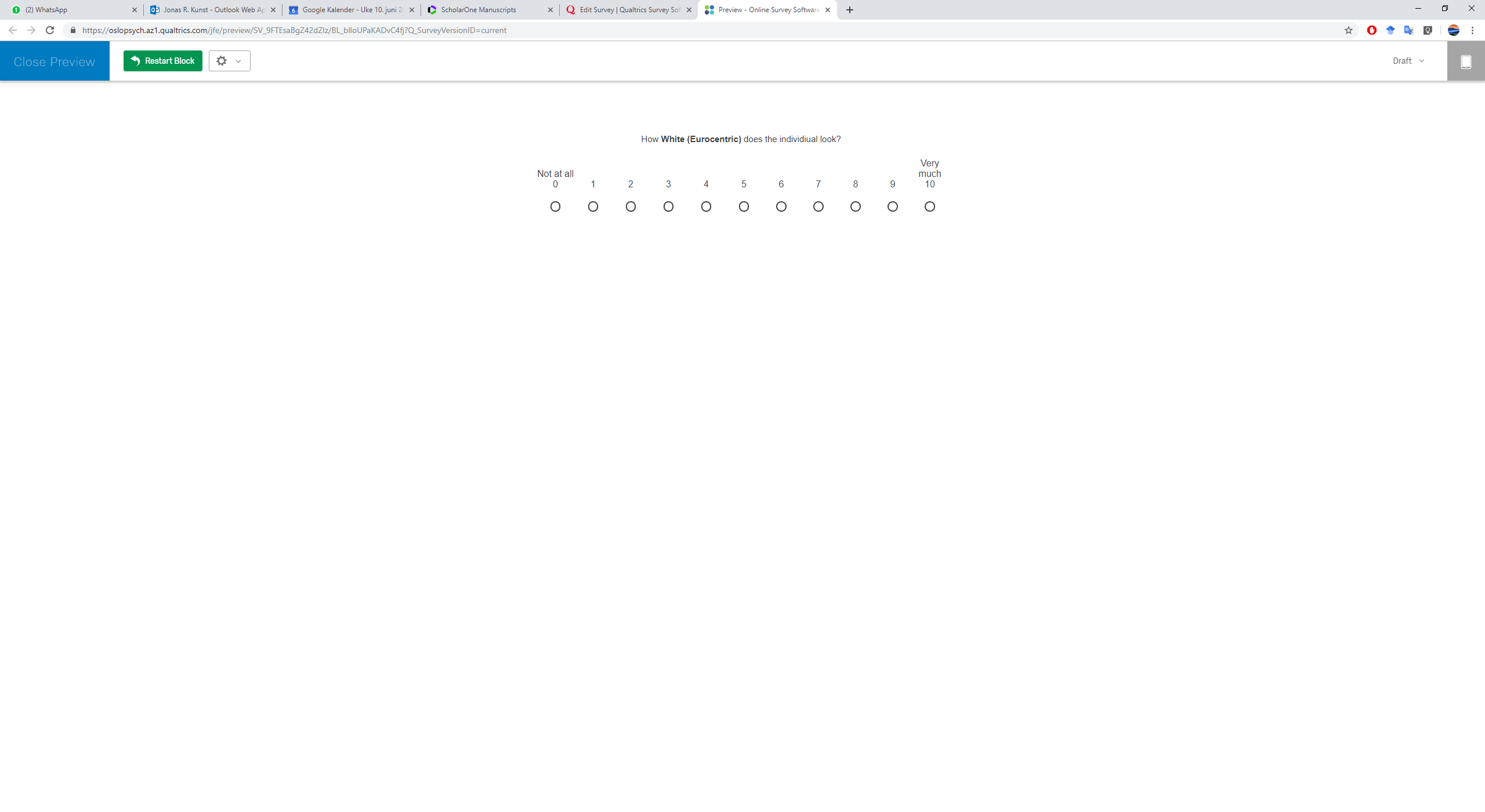


**[Warmth measure]**

**Please note:**
We will now ask you to rate how warm the individuals look.

[page break]

[image placeholder]

How **warm** does the individual look?


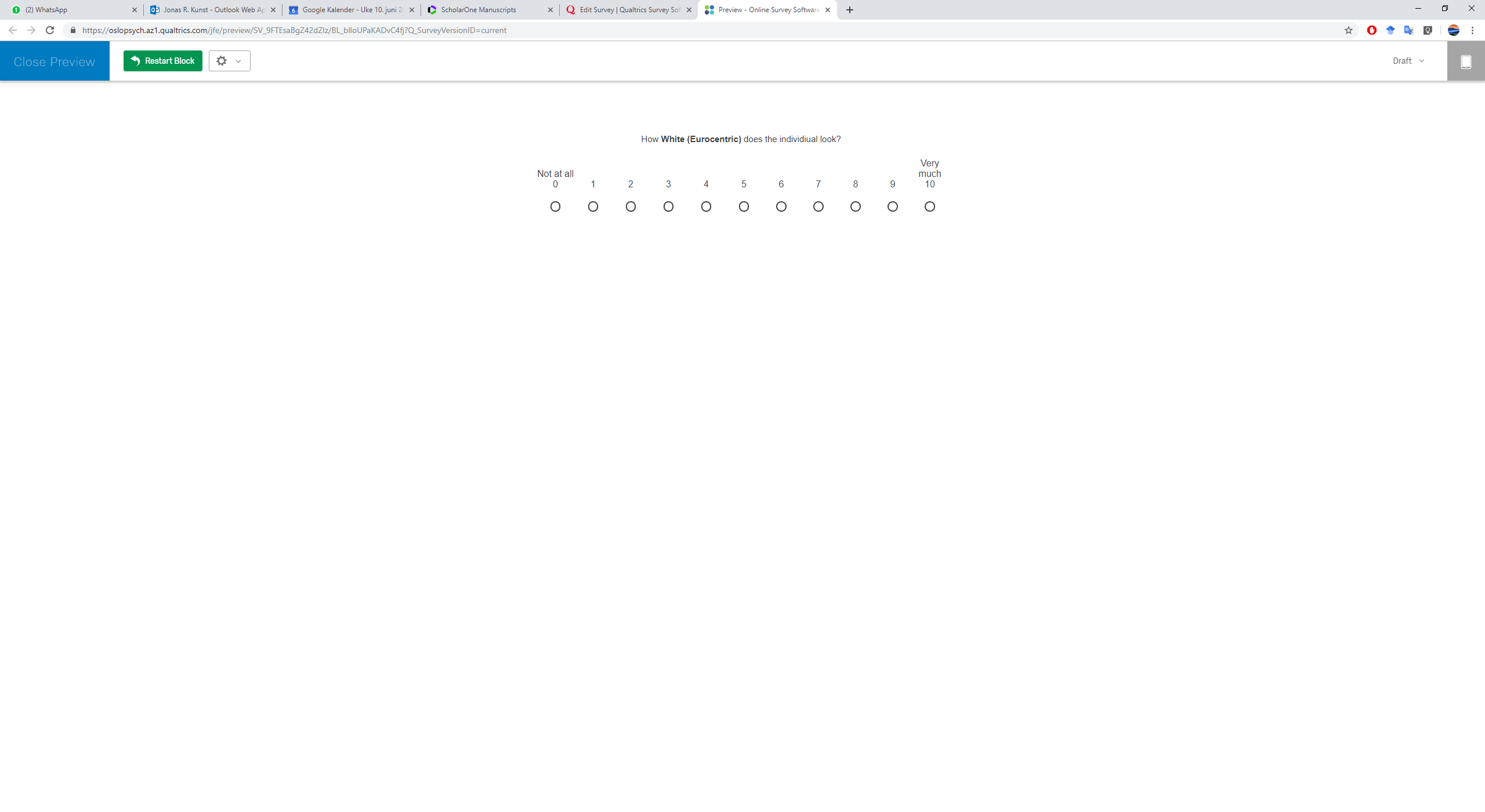


**[Competence measure]**

**Please note:**
We will now ask you to rate how competent the individuals look.

[page break]

[image placeholder]

How **competent** does the individual look?


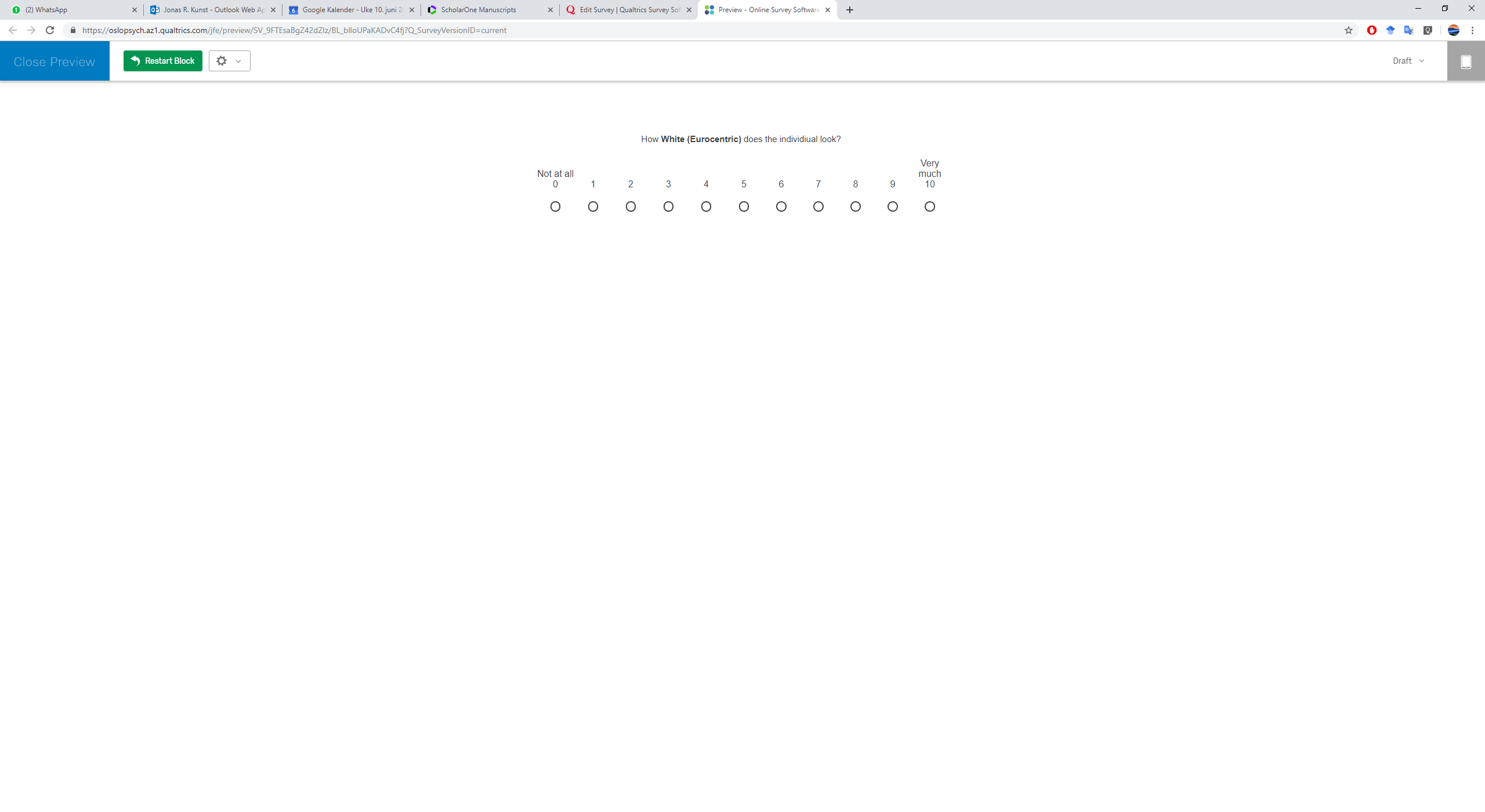


**[American measure]**

**Please note:**
We will now ask you to rate how American the individuals look.

[page break]

[image placeholder]

How **American** does the individual look?


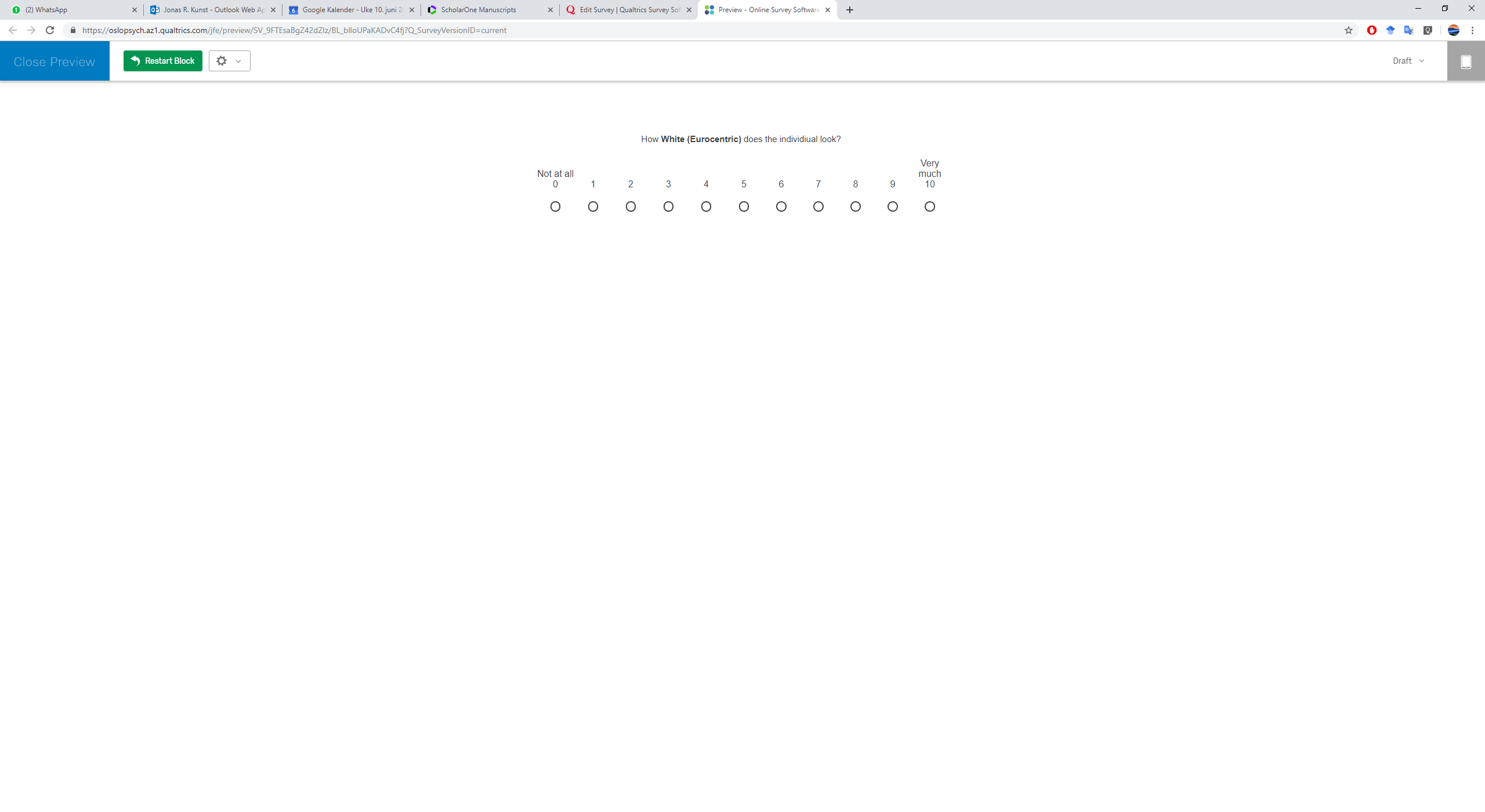


**[Social class measure]**

**Please note:**
We will now ask you to rate whether the individuals look lower class.

[page break]

[image placeholder]

How likely is it that the individual is **lower class**?


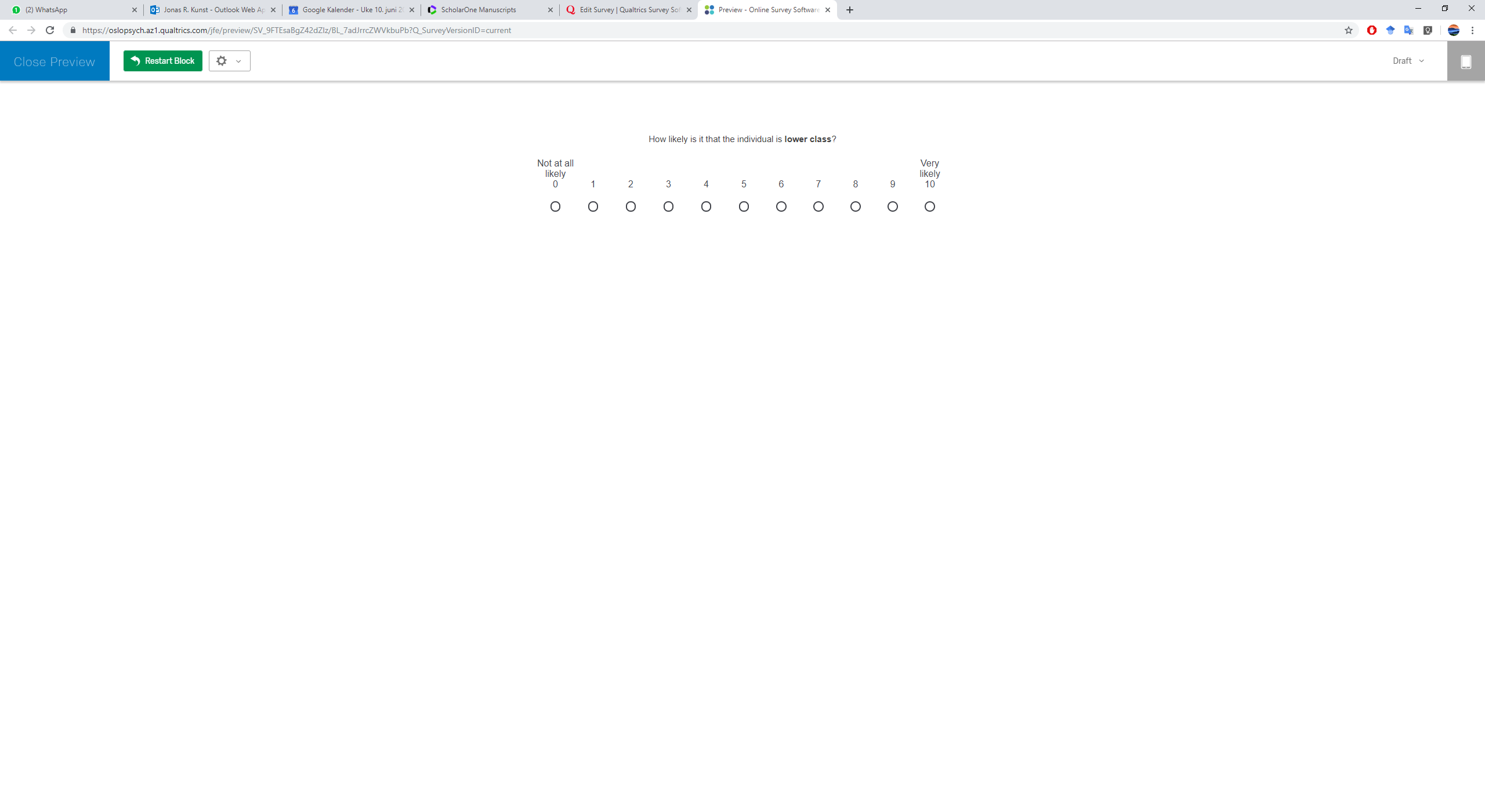


[page break]

You are more than halfway.
**Thank you for paying attention!**

[page break]

[Second set of measures.]

[All measures were presented in random order.]

**[Volunteer in Black Community]**

**Please note:**We will now ask you to rate how likely you think it is that the individuals would volunteer helping people addicted to drugs in predominantly **Black** communities.

[page break]

[image placeholder]

How likely is it that the individual would **volunteer helping drug addicts in Black communities**?
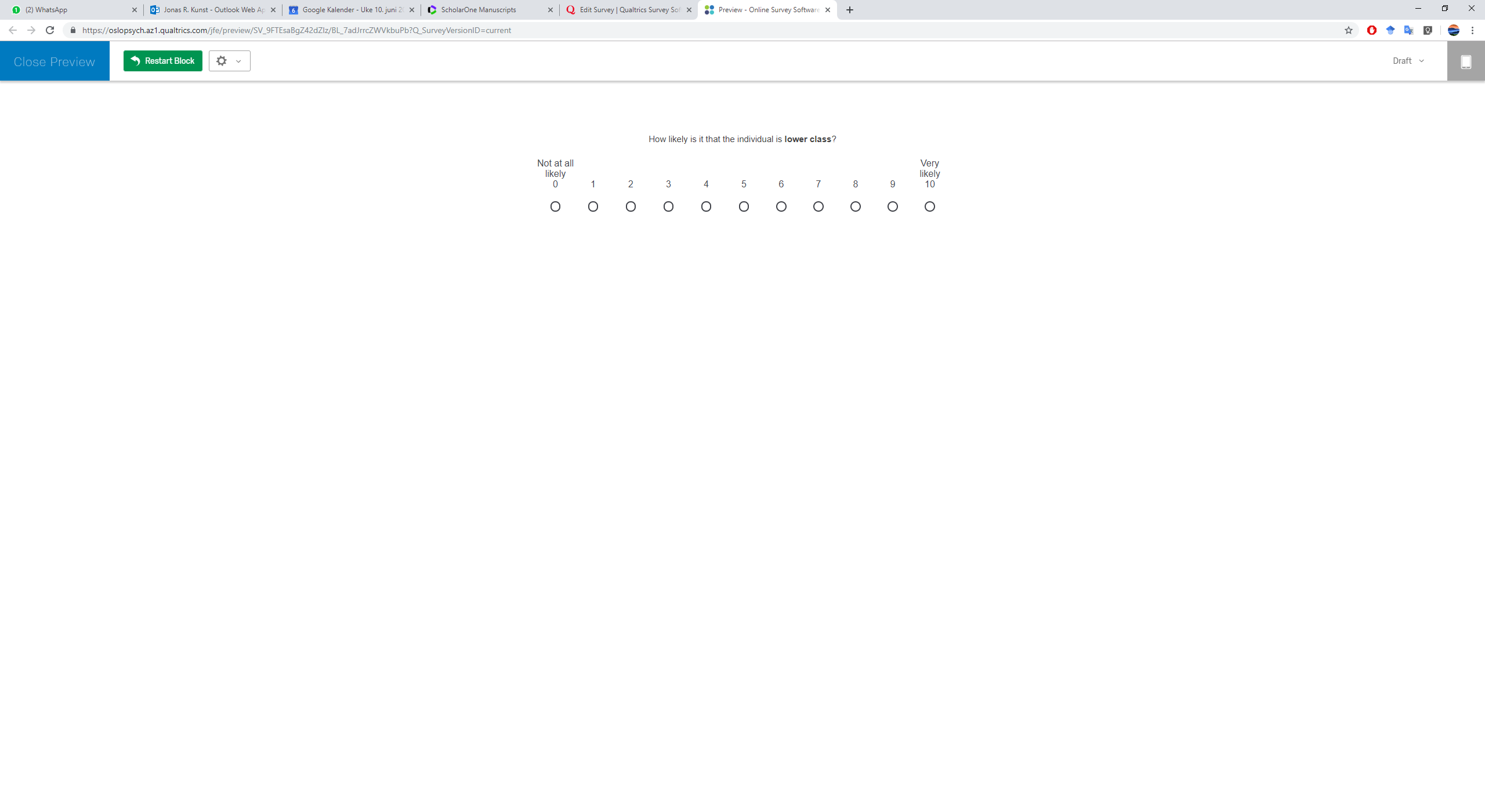


**[Support Black Lives Matter]**

**Please note:**We will now ask you to rate how likely you think it is that the individuals would participate in a Black Lives Matter march.

[page break]

[image placeholder]

How likely is it that the individual would participate in a **Black Lives Matter** march?
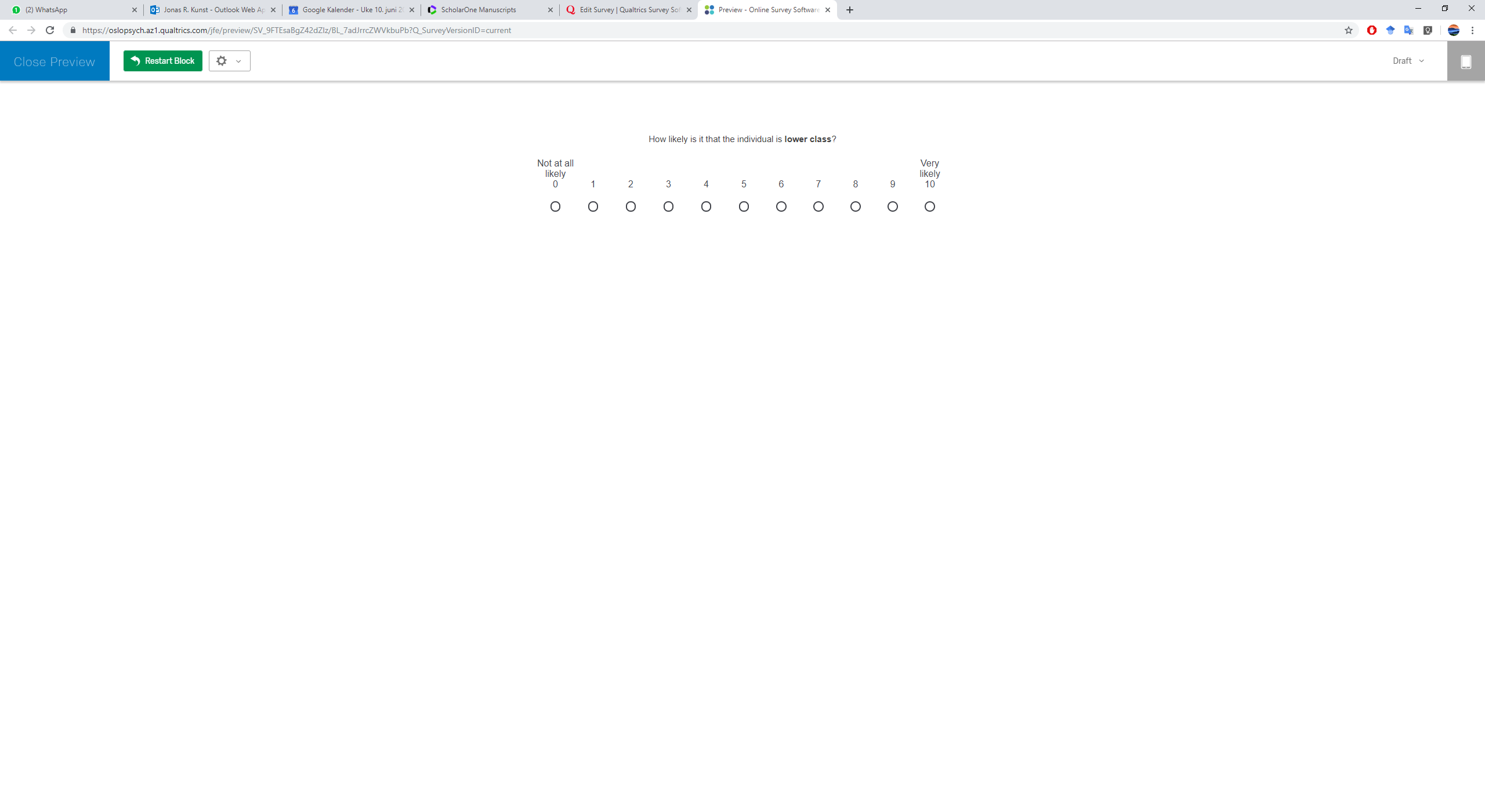


**[Volunteer in White Community]**

**Please note:**We will now ask you to rate how likely you think it is that the individuals would volunteer helping people addicted to drugs in predominantly **White** communities.

[page break]

[image placeholder]

How likely is it that the individual would **volunteer helping drug addicts in White communities**?
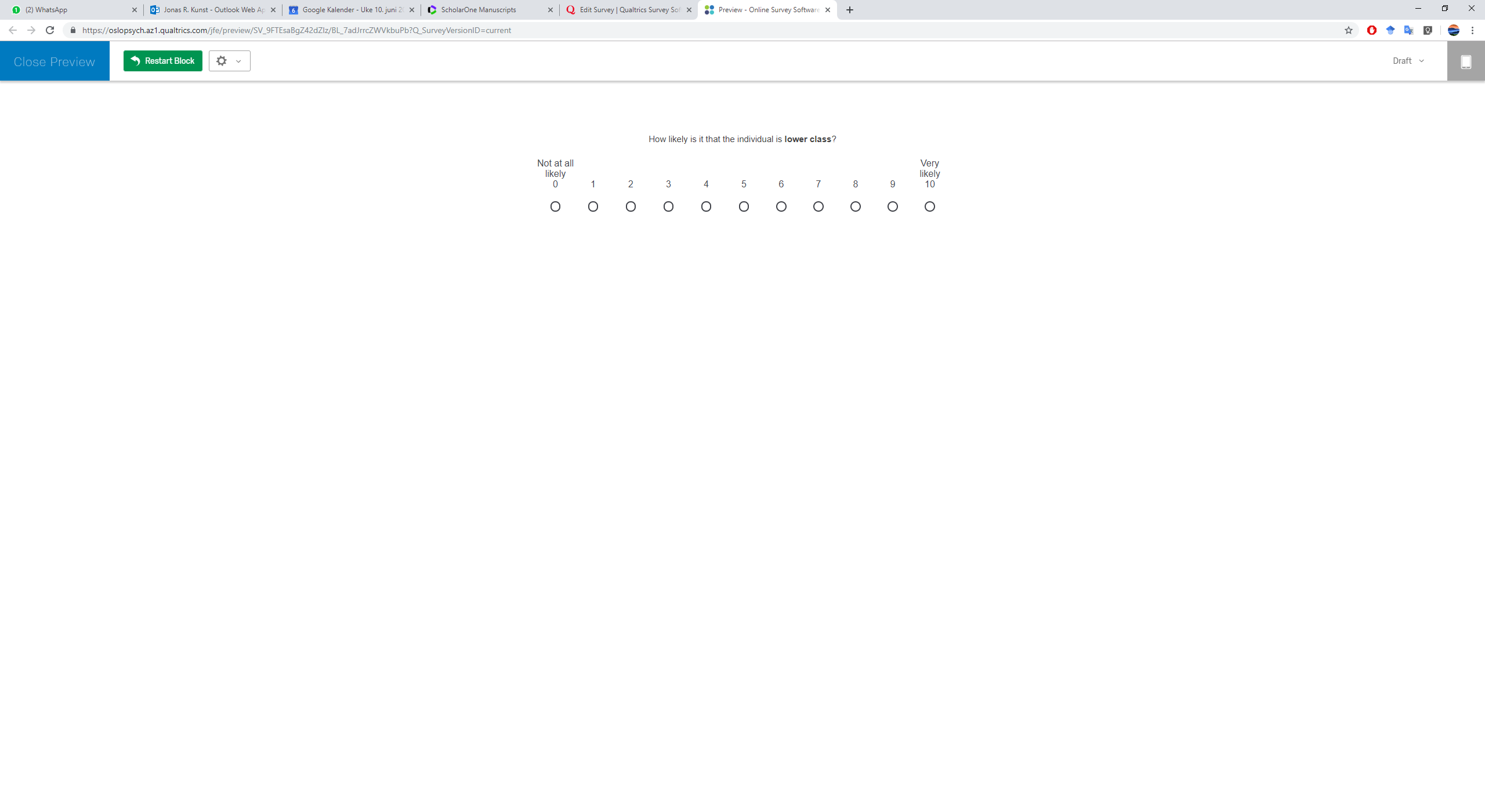


**[Support March Confederate Monuments]**

**Please note:**We will now ask you to rate how likely you think it is that the individuals would participate in a march for keeping confederate monuments.

[page break]

[image placeholder]

We will now ask you to rate how likely you think it is that the individuals would participate in a march for keeping confederate monuments.
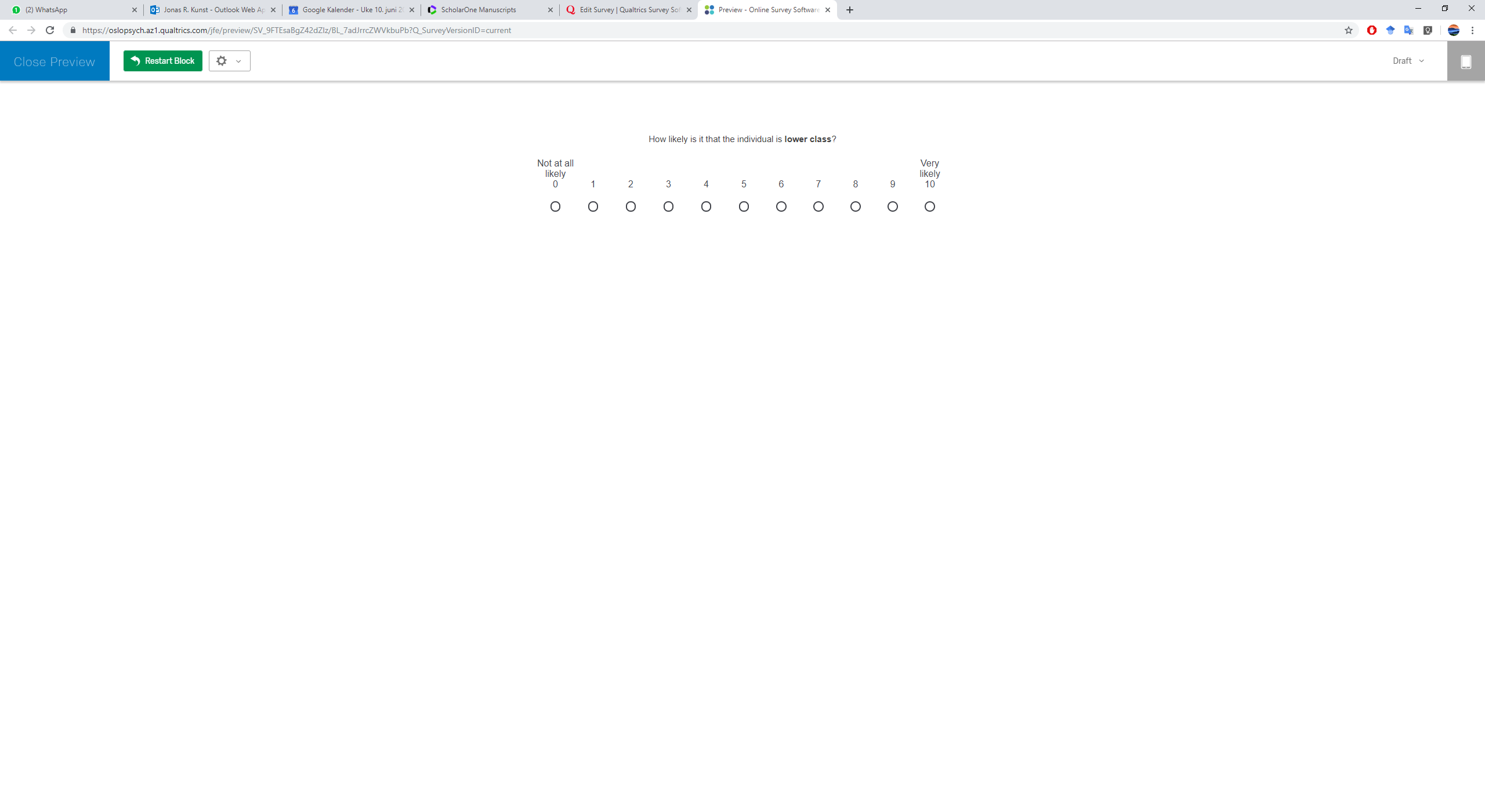


**[African American identification]**

**Please note:**
We will now ask you to rate how strongly you think the individuals identify with being African American.

[page break]

[image placeholder]

How strongly do you think the individual **identifies with being** **African American?**
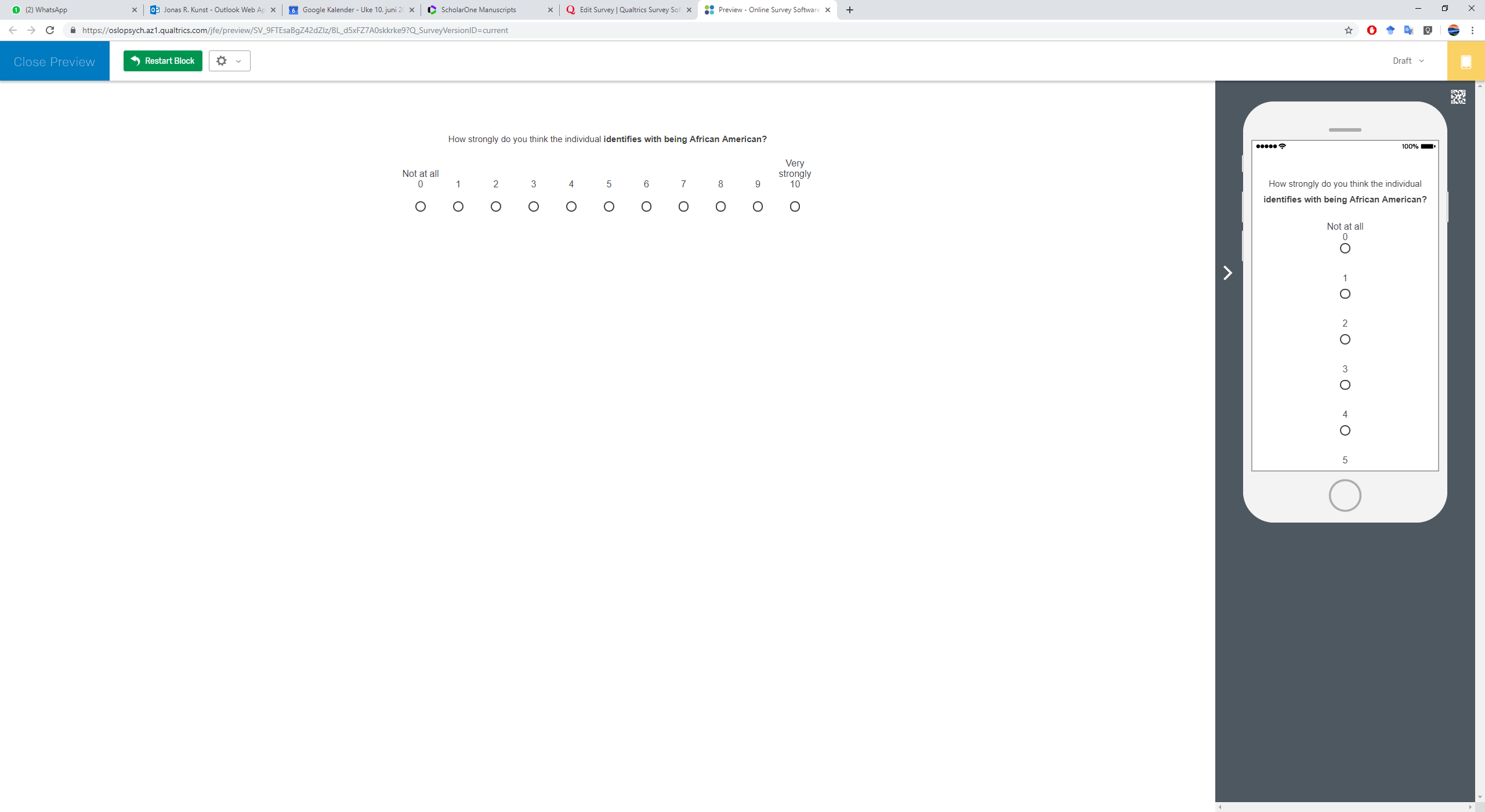


**[White American identification]**

**Please note:**
We will now ask you to rate how strongly you think the individuals identify with being White American.

[page break]

[image placeholder]

How strongly do you think the individual **identifies with being** **White American?**
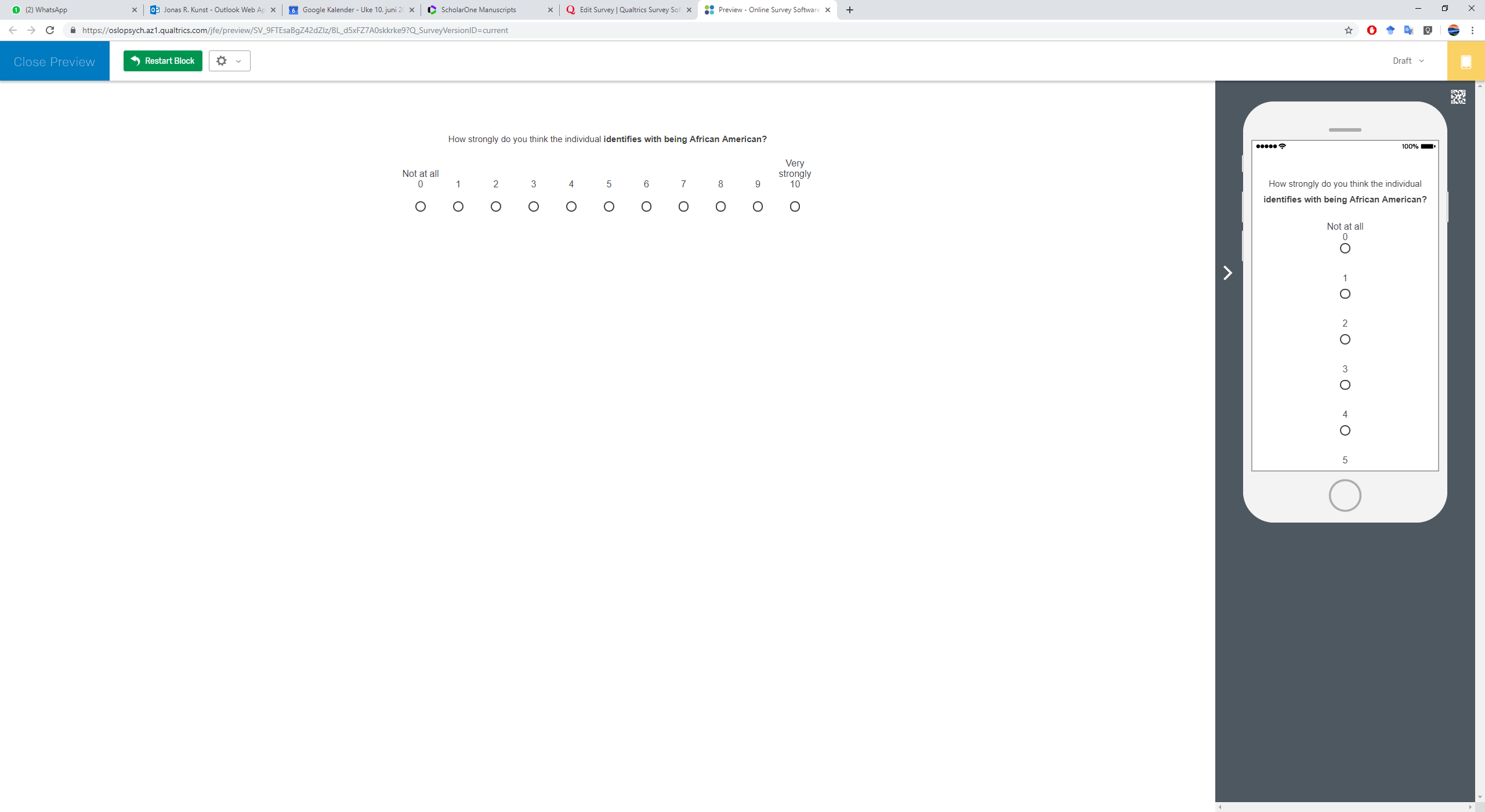

Supplement: sj-docx-1-psp-10.1177_01461672211024118 – Supplemental material for Knowledge About Individuals’ Interracial Friendships Is Systematically Associated With Mental Representations of Race, Traits, and Group Solidarity [file sj-docx-1-psp-10.1177_01461672211024118.docx]
